# NELL2, a novel osteoinductive factor, regulates osteoblast differentiation and bone homeostasis through fibronectin 1/integrin-mediated FAK/AKT signaling

**DOI:** 10.1038/s41413-025-00420-5

**Published:** 2025-04-11

**Authors:** Hairui Yuan, Xinyu Wang, Shuanglin Du, Mengyue Li, Endong Zhu, Jie Zhou, Yuan Dong, Shuang Wang, Liying Shan, Qian Liu, Baoli Wang

**Affiliations:** NHC Key Lab of Hormones and Development, Tianjin Key Lab of Metabolic Diseases, Chu Hsien-I Memorial Hospital & Institute of Endocrinology, Tianjin Medical University, Tianjin, 300134 China

**Keywords:** Bone, Osteoporosis

## Abstract

Neural EGFL-like 2 (NELL2) is a secreted protein known for its regulatory functions in the nervous and reproductive systems, yet its role in bone biology remains unexplored. In this study, we observed that NELL2 was diminished in the bone of aged and ovariectomized (OVX) mice, as well as in the serum of osteopenia and osteoporosis patients. In vitro loss-of-function and gain-of-function studies revealed that NELL2 facilitated osteoblast differentiation and impeded adipocyte differentiation from stromal progenitor cells. In vivo studies further demonstrated that the deletion of NELL2 in preosteoblasts resulted in decreased cancellous bone mass in mice. Mechanistically, NELL2 interacted with the FNI-type domain located at the C-terminus of Fibronectin 1 (Fn1). Moreover, we found that NELL2 activated the focal adhesion kinase (FAK)/AKT signaling pathway through Fn1/integrin β1 (ITGB1), leading to the promotion of osteogenesis and the inhibition of adipogenesis. Notably, administration of NELL2-AAV was found to ameliorate bone loss in OVX mice. These findings underscore the significant role of NELL2 in osteoblast differentiation and bone homeostasis, suggesting its potential as a therapeutic target for managing osteoporosis.

## Introduction

Osteoporosis is a prevalent bone disease affecting approximately 90 million individuals in China, primarily elderly and postmenopausal women.^1^ The pathogenesis of osteoporosis is fundamentally linked to an imbalance in bone remodeling, where osteoclast-driven bone resorption outpaces osteoblast-driven bone formation.^2^ Current therapeutic approaches for osteoporosis mainly focus on inhibiting bone resorption, whereas there are very few drugs available that promote bone formation. Additionally, none of these treatments are fully curative, and many are associated with significant side effects.^3^ Thus, gaining a deeper insight into the mechanisms underlying osteoblast differentiation and bone formation is essential for the development of therapeutic interventions to treat osteoporosis.

Osteoblasts originate from multiple cellular sources including bone marrow stromal cells (BMSCs), growth plate skeletal stem cells (GP-SSCs), and periosteal skeletal stem cells (P-SSCs).^4–6^ Regarding BMSCs, their heterogeneity has been extensively studied through single-cell transcriptomics, revealing distinct characteristics of the osteogenic and adipogenic subpopulations.^7,8^ Baccin et al. designated these two subpopulations as Osteo-CAR and Adipo-CAR, noting that they express osteogenic and adipocyte lineage genes, respectively, and are distinctly localized within the bone marrow.^8^ The differentiation of BMSCs is an orchestrated process regulated by various transcription factors and distinct signaling pathways.^9,10^ Key positive regulators of osteoblast differentiation include Runt-related transcription factor 2 (Runx2),^11^ osterix (Osx),^12^ as well as the Wnt/β-catenin and transforming growth factor-β (TGFβ)/bone morphogenetic proteins (BMPs) signaling pathways.^13–15^ In contrast, the major drivers of adipocyte differentiation include peroxisome proliferator-activated receptor γ (PPARγ) and the CCAAT/enhancer binding proteins (C/EBPs) family.^16^

Neural EGFL-like 2 (NELL2) is the homolog of Nel, which was initially identified from a chicken embryonic cDNA library.^17^ As a secreted glycoprotein, NELL2 is highly expressed in the hippocampus and cerebral cortex, regulating neuronal differentiation, axon guidance, spatial learning, sexual and feeding behavior.^18–22^ NELL2 is also expressed in postnatal testis, where it plays a role in sperm maturation.^23^ Structurally, NELL2 contains multiple functional domains, including an N-terminal thrombospondin-1 (TSP-1)-like module (TSPN), several EGF-repeats, von Willebrand factor C domains (vWFC), and N-terminal signaling peptides.^17^ Although the role of NELL1, another homolog of Nel, in mammalian skeletal tissues has been well-characterized,^24–27^ there is currently no literature reporting the involvement of NELL2 in regulating bone homeostasis.

In this study, we investigated the role of NELL2 in regulating osteoblast differentiation and bone homeostasis, and elucidated the underlying cellular and molecular mechanisms. The findings provide novel insights into the physiological significance of NELL2 as a crucial regulator in osteoblast differentiation and bone homeostasis, highlighting its potential as a therapeutic target for treating osteoporosis.

## Results

### NELL2 expression level was diminished in the bone of aged and OVX mice and in the serum of postmenopausal osteoporosis patients

We assessed the mRNA and protein expression of NELL2 in various tissues/organs from 6-week-old C57BL/6 J mice. NELL2 mRNA was predominantly observed in the brain and testis (Fig. S1a), while NELL2 protein was most abundant in the heart and brown adipose tissue, followed by the calvarial bone, long bone (tibia), kidney, muscle and brain (Fig. S1b). Given the observed expression of NELL2 in bone tissue, we further examined NELL2 expression in the tibiae of aged mice and ovariectomized (OVX) mice. The results revealed a downregulation of NELL2 protein in the tibiae of 18-month-old aged mice (Fig. S1c) and OVX mice (Fig. S1d) compared to 3-month-old young mice and sham-operated mice, respectively. We also collected the serum samples of postmenopausal women, and compared NELL2 levels among individuals with normal bone mass and those diagnosed with osteopenia or osteoporosis. The demographic and clinical characteristics of the participants were listed in Table S1. We found that serum levels of NELL2 were decreased in patients with osteopenia and osteoporosis compared to healthy individuals (Fig. S1e). Furthermore, we observed elevated NELL2 protein levels during osteogenic differentiation in stromal ST2 cells (Fig. S1f), suggesting a possible role of NELL2 in the regulation of osteogenesis.

### NELL2 promoted osteoblast differentiation and impeded adipocyte differentiation

**Fig. 1:**
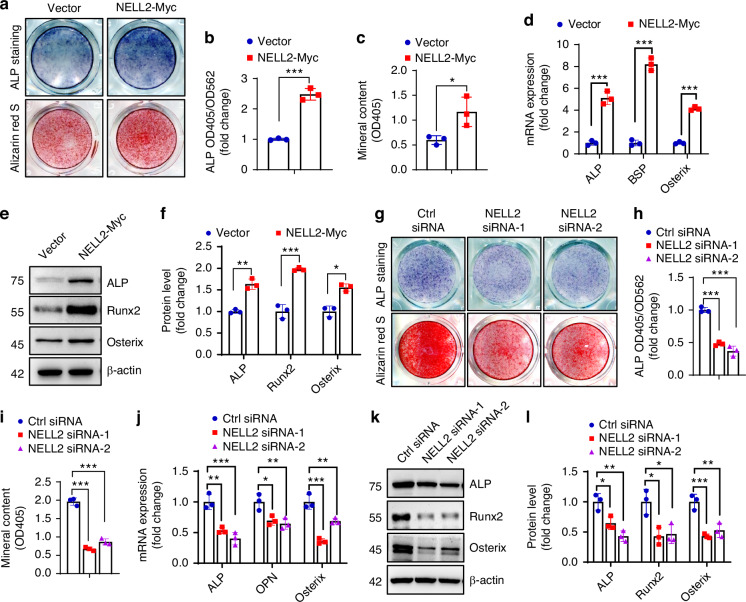
NELL2 promoted osteoblast differentiation. ST2 cells were cultured and induced to allow osteogenic differentiation after overexpression (**a**–**f**) or knockdown (**g**–**l**) of NELL2. **a**, **g** ALP staining (upper panel) and alizarin red S staining (lower panel) were performed after 14 and 21 days of induction, respectively. **b**, **h** ALP activity was measured. **c**, **i** The intensity of alizarin red S staining was quantified. **d**–**f**, **j**–**l** The mRNA (**d**, **j**) and protein (**e**, **f**, **k**, **l**) levels of osteogenic factors were detected after 3 days of induction. Data are mean ± SD, *n* = 3. Comparisons were conducted using Student’s *t* test (**b**–**d**, **f**), or one-way ANOVA followed by Dunnett’s test (**h**–**j**, **l**), *P < 0.05, **P < 0.01, ***P < 0.001

Compared with vector-transfected ST2 cells, overexpression of NELL2 enhanced alkaline phosphatase (ALP) staining, increased cellular ALP activity, and enhanced alizarin red S staining for matrix mineralization (Fig. S2a–c; Fig. 1a–c). Accordingly, the mRNA and protein levels of osteogenic factors such as Runx2, osterix, ALP and bone sialoprotein (BSP) were increased in NELL2-overexpressing cells after osteogenic treatment (Fig. 1d–f). Next, we used small-interfering RNAs (siRNAs) to silence endogenous NELL2 expression (Fig. S2i–k). Under osteogenic induction, silencing of NELL2 markedly suppressed osteoblast differentiation, as evidenced by the diminished ALP staining, alizarin red S staining and cellular ALP activity (Fig. 1g–i). Accordingly, the mRNA and protein levels of the key osteogenic factors were decreased in NELL2-silenced cells (Fig. 1j–l). Collectively, these findings indicate that NELL2 plays a role in promoting osteoblast differentiation.

We then investigated whether NELL2 regulates adipocyte differentiation. Our results showed that, under adipogenic induction, overexpression of NELL2 in ST2 cells significantly reduced differentiated oil red O-positive adipocytes (Fig. S2d, e), as well as the mRNA and protein expression of adipogenic markers such as PPARγ, C/EBPα, and adipocyte protein 2 (aP2) in ST2 cells compared to Vector (Fig. S2f–h). In contrast, knockdown of NELL2 promoted adipogenic differentiation (Fig. S2l–p). Taken together, these data suggest that NELL2 promotes osteoblast differentiation and inhibits adipocyte differentiation from stromal progenitor cells.

### Preosteoblast-specific deletion of NELL2 reduced bone mass in mice

To investigate the physiological function of NELL2 in bone, we generated Col1-cre;*Nell2*^fx/fx^ in which NELL2 was deleted in preosteoblasts. We confirmed the deletion efficiency in calvarial preosteoblasts using quantitative reverse transcription polymerase chain reaction (qRT-PCR) and western blotting (Fig. S3a–c). We found that the knockout of NELL2 in preosteoblasts does not alter the expression level of NELL1 (Fig. S3d). Three-month-old Col1-cre;*Nell2*^fx/fx^ mice showed no significant differences in body weight and length compared to the *Nell2*^fx/fx^ mice (Fig. S3e, f, j, k). μCT analysis revealed that the cortical bone thickness (Ct. Th) and cortical bone mineral density (Ct. BMD) of the tibiae in 3-month-old Col1-cre;*Nell2*^fx/fx^ mice did not show significant difference compared with *Nell2*^fx/fx^ mice (Fig. S3g–i, l–n). Alcian Blue/Hematoxylin/Orange G (ABH/OG) staining revealed no significant changes in growth plate morphology in male and female mice (Fig. S3o).

**Fig. 2:**
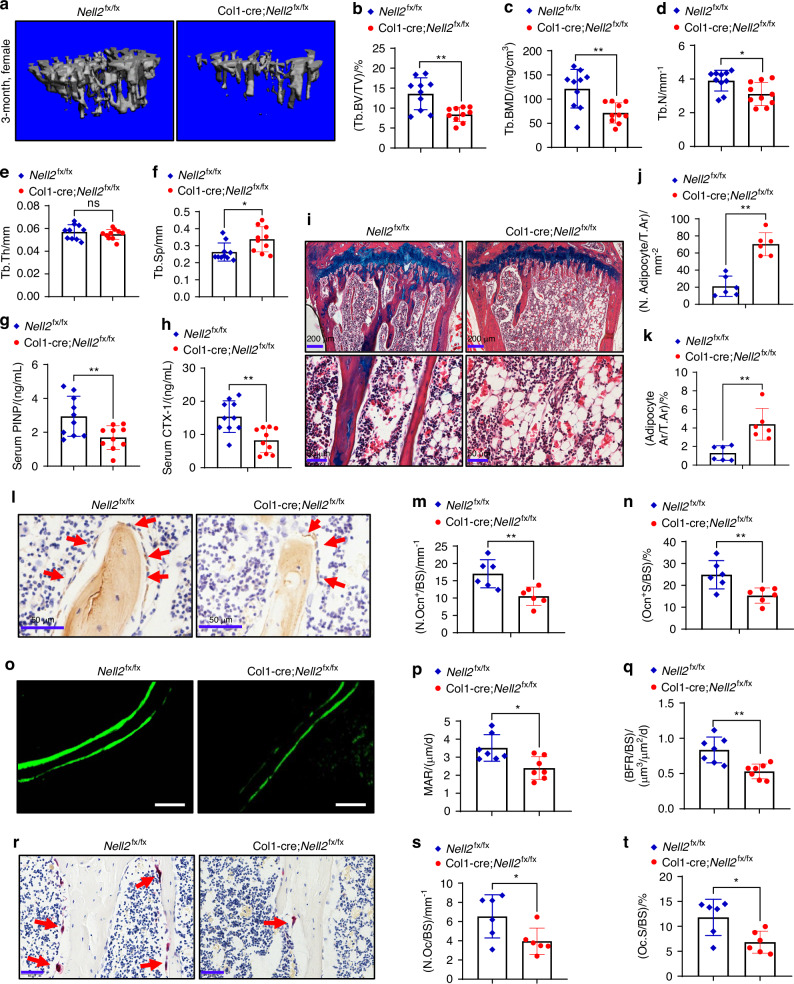
Preosteoblast-specific deletion of NELL2 reduced bone mass in female mice. **a**–**f** Cancellous bone mass at proximal tibial metaphysis in 3-month-old female mice was analyzed using μCT. **a** Representative reconstruction images are shown. **b**–**f** Histomorphometric parameters of cancellous bone were quantified, *n* = 10. **g**, **h** Serum levels of PINP and CTX-1 were measured using ELISA, *n* = 10. **i** ABH/OG staining was conducted. Scale bar: 200 μm (upper panel); 50 μm (lower panel). **j**, **k** The numbers and area percentage of adipocytes were quantified, *n* = 6. **l** Representative images of osteocalcin IHC staining are shown. **m**, **n** The numbers and surface percentage of trabecular Ocn^+^ osteoblasts were quantified, *n* = 6. **o** Representative images of calcein double labeling are shown. **p**, **q** MAR and BFR/BS were quantified, *n* = 7. **r** Representative images of TRAP staining are shown. **s**, **t** The numbers and surface percentage of osteoclasts were quantified, *n* = 6. Scale in (**l**, **o**, **r**): 50 μm. Data are mean ± SD. Comparisons were conducted using Student’s *t* test, *P < 0.05, **P < 0.01; ns: no significance

However, the cancellous bone mass at the metaphysis was decreased in 3-month-old Col1-cre;*Nell2*^fx/fx^ female mice. In brief, trabecular bone volume/total volume (Tb. BV/TV), trabecular bone mineral density (Tb. BMD), and trabecular number (Tb. N) were reduced by 38.1%, 41.2% and 20.3%, respectively, while trabecular separation (Tb. Sp) was increased by 28.5% in 3-month-old Col1-cre;*Nell2*^fx/fx^ female mice versus *Nell2*^fx/fx^ mice (Fig. 2a–f). Similar results were observed in 3-month-old male mice (Fig. S4a–f). In both genders, Col1-cre;*Nell2*^fx/fx^ mice exhibited a decrease in serum levels of procollagen type I N-terminal propeptide (PINP) and C-terminal telopeptide of type I collagen (CTX-1), markers for bone formation and resorption, respectively (Fig. 2g, h; Fig. S4g, h), indicating a diminished bone turnover rate due to NELL2 deletion in preosteoblasts.

ABH/OG staining showed that there was an increase in the number and area percentage of adipocytes in the bone marrow compartment (Fig. 2i–k; Fig. S4i–k). Immunohistochemistry (IHC) results showed a decrease in the number and surface percentage of osteocalcin (Ocn)-positive osteoblasts (Fig. 2l–n; Fig. S4l–n). Calcein double-labeling analysis revealed that Col1-cre;*Nell2*^fx/fx^ female mice had lower mineral apposition rate (MAR) and bone formation rate/bone surface (BFR/BS) compared with *Nell2*^fx/fx^ mice (Fig. 2o–q). Tartrate-resistant acid phosphatase (TRAP) staining revealed a decrease in the number and surface percentage of TRAP^+^ osteoclasts in Col1-cre;*Nell2*^fx/fx^ mice (Fig. 2r–t; Fig. S4o–q). qRT-PCR analysis demonstrated a downregulation of the Rankl/opg ratio (Fig. S5a–c) in preosteoblasts from Col1-cre;*Nell2*^fx/fx^ mice, suggesting that NELL2 expressed in osteoblastic lineage may regulate osteoclastogenesis. Furthermore, impaired osteoclast differentiation was observed in bone marrow cells derived from Col1-cre;*Nell2*^fx/fx^ mice compared to those from control mice, as indicated by a decrease in the number of osteoclasts formed and downregulation of osteoclast-specific genes such as acid phosphatase 5 (Acp5), cathepsin K (Ctsk) and nuclear factor of activated T cells 1 (Nfatc1) (Fig. S5d–f).

### NELL2 deletion in preosteoblasts reduced osteoblast differentiation and promoted adipocyte differentiation

**Fig. 3:**
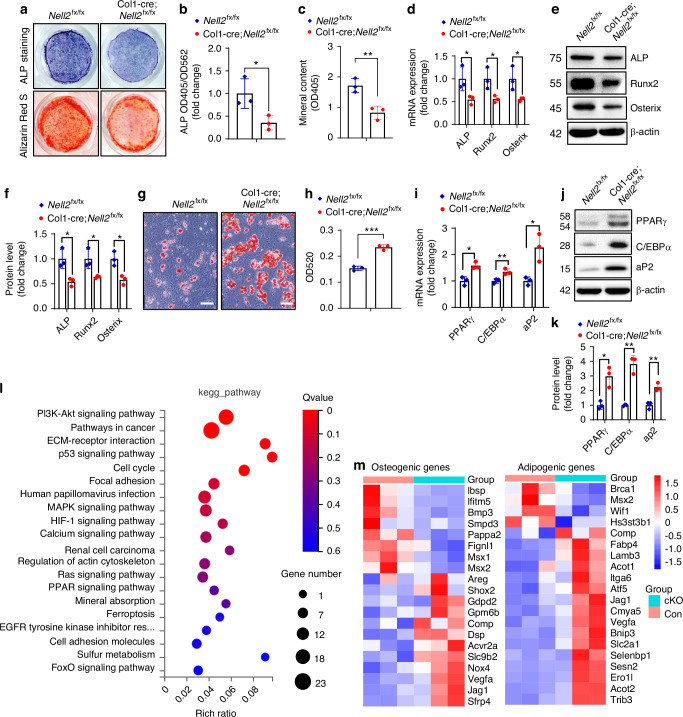
NELL2 deletion in preosteoblasts reduced osteoblast differentiation and promoted adipocyte differentiation. Primary calvarial preosteoblasts from mice were cultured and induced to allow osteogenic (**a**–**f**) and adipogenic (**g**–**k**) differentiation. **a** ALP staining and alizarin red S staining were conducted after 14 and 21 days of induction, respectively. **b** ALP activity was measured. **c** The intensity of alizarin red S staining was quantified. **d**–**f** The mRNA and protein levels of osteogenic factors were determined after 3 days of induction. **g** Oil red O staining was conducted. Scale bar: 100 μm. **h** The intensity of oil red O staining was quantified by measuring OD520. **i**–**k** The mRNA and protein levels of adipogenic factors were examined following induction for 2 and 3 days respectively. **l** KEGG analysis was performed to identify the top-ranking enriched pathways. **m** A heatmap illustrating differentially expressed osteogenic and adipogenic genes is presented. Data are mean ± SD, *n* = 3. Comparisons were conducted using Student’s *t* test, *P < 0.05, **P < 0.01, ***P < 0.001

To further validate the physiological role of NELL2, we examined the differentiation potential of neonatal calvarial preosteoblasts from the mouse models. For osteoblast differentiation, preosteoblasts from Col1-cre;*Nell2*^fx/fx^ mice exhibited diminished ALP staining, alizarin red S staining and cellular ALP activity, as well as downregulation of osteogenic factors compared with *Nell2*^fx/fx^ controls (Fig. 3a–f). For adipogenic differentiation, preosteoblasts from Col1-cre;*Nell2*^fx/fx^ mice showed enhanced ability to differentiate into adipocytes, manifested by enhanced oil red O staining and upregulation of adipogenic factors (Fig. 3g–k). These findings indicated that the deletion of endogenous NELL2 inhibited osteoblast differentiation and mineralization, and promoted adipocyte formation.

The expression profiles in preosteoblasts between the mutants and controls were compared by RNA-seq analysis (GSE266198). The results revealed a total of 311 differentially expressed genes, with 107 genes downregulated and 204 genes upregulated (Fig. S6). Kyoto encyclopedia of genes and genomes (KEGG) analysis indicated that these genes are involved in pathways such as phosphatidylinositol 3-kinase (PI3K)-Akt, cancer, extracellular matrix (ECM)-receptor interaction, p53, cell cycle, focal adhesion, etc (Fig. 3l). Gene ontology (GO) analysis revealed that the deletion of NELL2 resulted in expression changes in multiple genes implicated in osteoblast and adipocyte differentiation (Fig. 3m).

### NELL2 interacted with Fibronectin 1 in BMSCs

**Fig. 4:**
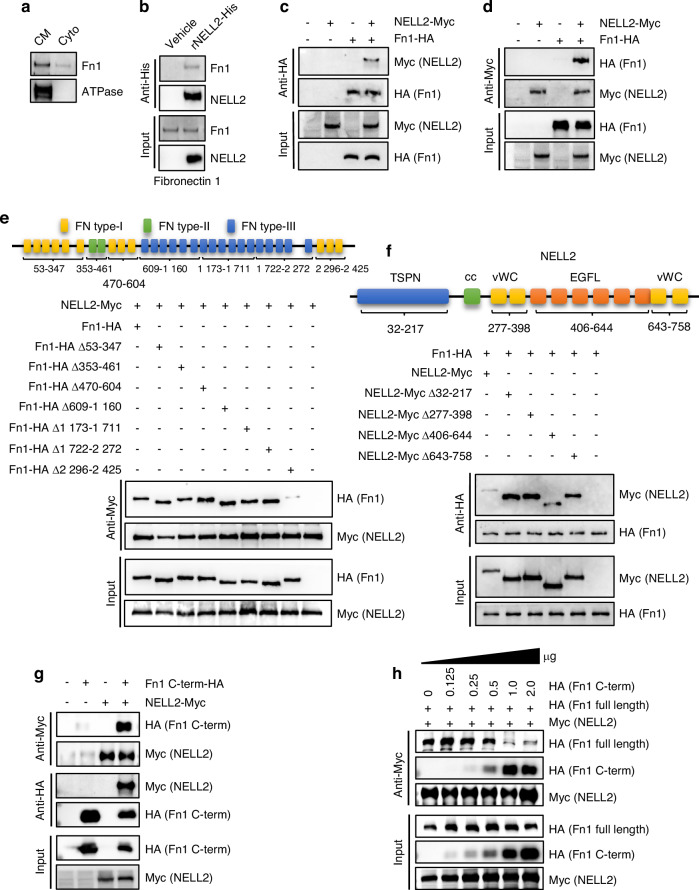
NELL2 interacted with Fibronectin 1 in BMSCs. **a** The membrane location of Fn1 in BMSCs was validated using western blotting. **b** The presence of Fn1 in the membrane-bound proteins pulled down by NELL2-His protein was confirmed using Western blotting. **c**, **d** NELL2-Myc and Fn1-HA expression constructs were co-transfected into 293 T cells and Co-IP was performed to confirm the binding of NELL2-Myc to Fn1-HA. **e** Co-IP was performed to analyze the binding of various Fn1 truncates to full-length NELL2. **f** Co-IP was performed to analyze the binding of various NELL2-Myc truncates to full-length Fn1-HA. **g** Co-IP was performed to analyze the interaction between NELL2-Myc with Fn1 C-terminal-HA fragment. **h** NELL2-Myc (1 μg) and Fn1-HA (1 μg) expression constructs were co-transfected into 293 T cells, along with a quantity gradient of 0–2 μg of Fn1 C-terminal-HA construct. Immunoprecipitation was performed using anti-Myc, followed by Western blotting analysis using anti-HA and anti-Myc

The reported receptors for NELL2 include roundabout guidance receptor 2/3 (ROBO2/3) and c-ros oncogene 1 (ROS1),^22,23^ neither of which was expressed or expressed at very low levels in preosteoblasts as indicated in our RNA-seq data. We therefore attempted to screen for the potential receptors or binding partners through which NELL2 signals in BMSCs using pull-down assay followed by liquid chromatography-tandem mass spectrometry (LC-MS/MS). Our results revealed a total of 173 proteins pulled down, including Fn1 ranking at top 1 (Table S2). We confirmed that Fn1 is primarily located on cell membrane (Fig. 4a). We further validated the MS results and demonstrated that NELL2-His recombinant protein indeed interacted with Fn1 (Fig. 4b). To further demonstrate the direct interaction between NELL2 and Fn1, we co-transfected NELL2-Myc and Fn1-HA into 293 T cells, and collected the cell lysates for Co-immunoprecipitation (Co-IP). Western blotting detected a strong band of Myc-tagged protein in HA immunoprecipitates (Fig. 4c) and a strong band of HA-tagged protein in Myc immunoprecipitates (Fig. 4d).

Next, we conducted sequential deletions to generate a series of Fn1 truncation mutants that lack specific domains including FNI (53-347 aa), FNII (353-461 aa), FNI (470-604 aa), FNIII 1-6 (609-1160 aa), FNIII 7-12 (1173-1711 aa), FNIII 13-17 (1722-2272 aa), and FNI (2296-2425 aa). Each mutant construct was co-transfected with NELL2-Myc construct into 293 T cells. Co-IP experiments using anti-Myc antibody showed that the deletion of FNI (2296-2425 aa) resulted in diminished binding between NELL2 and Fn1 (Fig. 4e). Additionally, we also generated a series of NELL2 constructs with sequential deletions in the domains of TSPN (32-217 aa), Coiled coil (242-271 aa), vWC (277-398 aa), EGF-like (406-644 aa), and vWC (643-758 aa), respectively. Co-IP experiments using anti-HA revealed that deletion of the EGF-like (406-644 aa) domain in NELL2 reduced the binding between Fn1 and NELL2 (Fig. 4f). These findings indicate that NELL2 interacts with Fn1 primarily through the FNI (2296-2425 aa) domain of Fn1 and the EGF-like domain of NELL2 (406-644 aa).

Furthermore, we tested whether the FNI (2296-2425 aa) domain of Fn1 interacts with NELL2. We co-transfected Fn1 C-terminal-HA construct (expresses FNI 2296-2425 aa domain of Fn1) and NELL2-Myc construct into 293 T cells. Co-IP with either Myc or HA beads followed by western blotting with HA or Myc antibody, respectively, showed a clear interaction between Fn1 C-terminal-HA and NELL2-Myc (Fig. 4g).

### NELL2 regulated osteoblast and adipocyte differentiation via interacting with Fibronectin 1

To determine whether Fn1 is involved in osteoblast and adipocyte differentiation, we utilized two independent siRNAs to silence endogenous Fn1 expression (Fig. S7a). The knockdown of Fn1 suppressed osteogenic differentiation in ST2, as evidenced by diminished ALP staining and cellular ALP activity (Fig. S7b, c), as well as downregulated expression of osteogenic factors (Fig. S7d–f). Conversely, the knockdown of Fn1 promoted adipogenic differentiation in ST2 cells (Fig. S8a–e). To further validate whether NELL2 regulates osteoblast and adipocyte differentiation through Fn1, we conducted co-transfection experiments with NELL2-Myc expression construct and Fn1 siRNA. The results demonstrated that the stimulatory effect of NELL2 on osteoblast differentiation was attenuated upon co-transfection with Fn1 siRNA (Fig. S7g–m). Similarly, the inhibitory effect of NELL2 on adipocyte differentiation was mitigated when co-transfected with Fn1 siRNA (Fig. S8f–j).

**Fig. 5:**
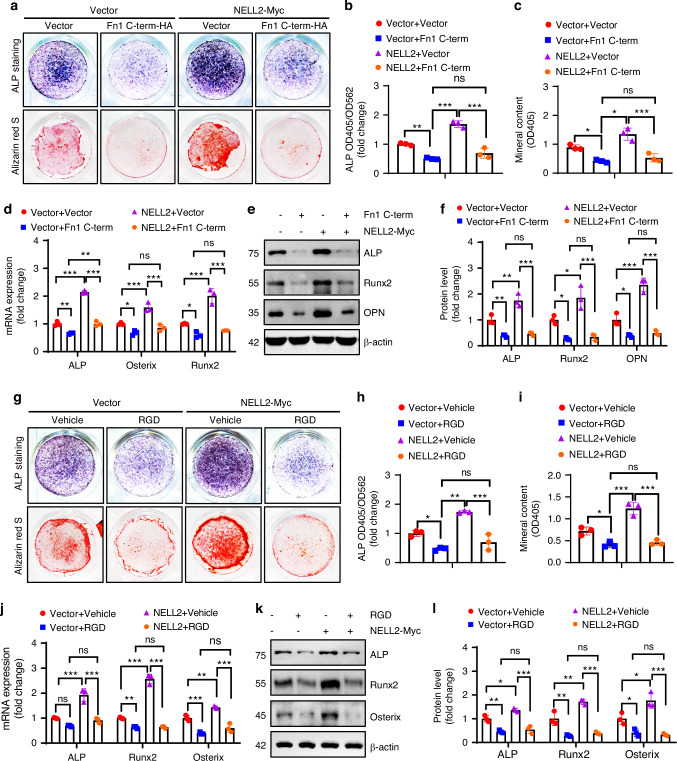
NELL2 regulated osteoblast differentiation through interacting with Fibronectin 1/integrin. **a**–**f** ST2 cells were co-transfected with NELL2-Myc (or vector) and Fn1 C-terminal-HA constructs (or vector), and then induced to allow osteogenic differentiation. **g**–**l** ST2 cells were transfected with NELL2-Myc, and then induced to allow osteogenic differentiation in the presence of vehicle or 10 μmol/L RGD peptide (GRGDSPK). **a**, **g** ALP staining (upper panel) and alizarin red S staining (lower panel) were performed after 14 and 21 days of induction, respectively. **b**, **h** ALP activity was measured, *n* = 3. **c**, **i** The intensity of alizarin red S staining was quantified, *n* = 3. **d**–**f**, **j**–**l** The mRNA (**d**, **j**) and protein (**e**, **f**, **k**, **l**) levels of osteogenic factors were detected after 3 days of induction, *n* = 3. Data are mean ± SD. Comparisons were conducted using two-way ANOVA followed by Tukey’s test, *P < 0.05, **P < 0.01, ***P < 0.001; ns no significance

To further investigate the necessity of NELL2-Fn1 interaction for osteoblast and adipocyte differentiation, 293 T cells were co-transfected using NELL2-Myc and full-length Fn1-HA along with various amounts of Fn1 C-terminal-HA. The Co-IP experiment showed that when NELL2-Myc was pulled down with anti-Myc, the amount of co-precipitated full-length Fn1-HA decreased as the amount of co-precipitated Fn1 C-terminal-HA increased (Fig. 4h). This indicates that an excess of Fn1 C-terminal fragment antagonized the binding of NELL2 to the full-length Fn1. We co-transfected ST2 cells with Fn1 C-terminal-HA and NELL2-Myc constructs, followed by induction of osteogenesis or adipogenesis. Notably, overexpression of Fn1 C-terminal-HA largely mitigated the pro-osteogenic and anti-adipogenic effects of NELL2 (Fig. 5a–f; Fig. S9a–g). These findings indicate that NELL2 regulated osteoblast and adipocyte differentiation via interacting with Fn1.

### NELL2 activated FAK/AKT signaling pathway through Fibronectin 1/Integrin β1

To elucidate the signaling pathway downstream of NELL2/Fn1, we conducted RNA-seq analysis using ST2 cells transfected with either NELL2 construct or Fn1 siRNA (GSE265936). The results revealed that NELL2 overexpression led to upregulation of 371 genes and downregulation of 293 genes (Fig. S10a), whereas Fn1 knockdown resulted in upregulation of 516 genes and downregulation of 812 genes (Fig. S10b). Venn diagram analysis identified a total of 236 genes changed in both NELL2-transfected and Fn1 siRNA-transfected cells (Fig. S10c). Subsequent pathway analysis of the overlapping altered genes showed that both NELL2 and Fn1 regulated actin cytoskeleton, focal adhesion, PI3K-Akt, cell adhesion pathways, etc (Fig. S10d). These pathways are primarily associated with integrins, which connect extracellular matrix (ECM) molecules to intracellular cytoskeletal proteins forming focal adhesions and mediating cell adhesion and bidirectional signaling between cells and ECM.^28^

We next investigated whether NELL2 regulates osteoblast and adipocyte differentiation through Fn1/integrin. To disrupt the fibronectin-integrin interaction, we employed GRGDSPK, a peptide that includes the Arg-Gly-Asp (RGD) sequence, which serves as a competitive and reversible inhibitor of fibronectin-integrin binding. Our results showed that treatment of ST2 cells with RGD peptide resulted in decreased osteogenesis and enhanced adipogenesis. Notably, the effects of NELL2 on osteoblast and adipocyte differentiation were largely attenuated in the presence of RGD peptide (Fig. 5g–l; Fig. S11a–f). These findings indicate that RGD peptide, by competitively binding the integrin receptor, mitigates NELL2-induced promotion of osteoblast differentiation and inhibition of adipocyte differentiation.

Integrin β1 (ITGB1) is known as the receptor of Fn1. Therefore, we determined whether ITGB1 is involved in the NELL2-regulated osteoblast and adipocyte differentiation. NELL2-Myc was transfected into ST2 cells with or without Fn1 siRNA. Co-IP experiment using anti-Myc antibody revealed that knockdown of Fn1 reduced the binding of Fn1 to NELL2, as well as the binding of NELL2 to ITGB1 (Fig. S12), suggesting that Fn1 is required for NELL2’s interaction with ITGB1. Subsequently, we transfected ST2 cells with ITGB1 siRNA, which led to a 95% reduction in ITGB1 mRNA level (Fig. S13a, b). Knockdown of ITGB1 inhibited osteoblast differentiation while promoting adipocyte differentiation in ST2 cells. Additionally, knockdown of ITGB largely mitigated the pro-osteogenic and anti-adipogenic effects of NELL2 (Fig. S13c–m). These results underscore the necessity of integrin for the functional role of NELL2.

**Fig. 6:**
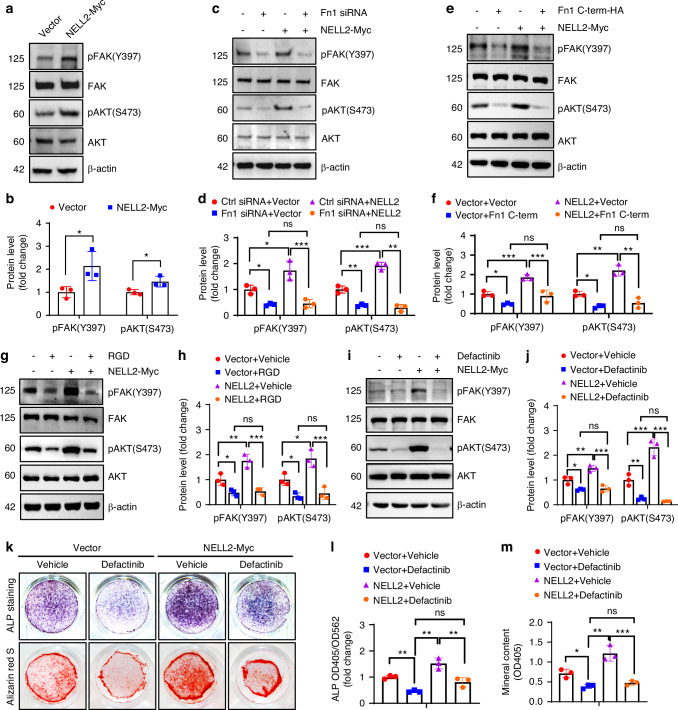
NELL2 activated FAK/AKT signaling pathway through Fibronectin 1/integrin. **a**–**j** Western blotting was conducted to analyze pFAK(Y397) and pAKT(S473) levels in ST2 cells either transfected with NELL2-Myc construct (or vector) (**a**, **b**), Fn1 siRNAs (or control siRNA) (**c**, **d**) or Fn1 C-terminal-HA (or vector) (**e**, **f**), or treated with RGD peptide (or vehicle) (**g**, **h**) or Defactinib (or vehicle) (**i**, **j**). **k**–**m** ST2 cells were transfected with NELL2-Myc (or vector), and then induced to allow osteogenic differentiation in the presence of Vehicle or 1 μmol/L Defactinib. **k** ALP staining (upper panel) and alizarin red S staining (lower panel) were performed after 14 and 21 days of induction, respectively. **l** ALP activity was measured. **m** The intensity of alizarin red S staining was quantified. Comparisons were conducted using Student’s *t* test (**b**), or two-way ANOVA followed by Tukey’s test (**d**, **f**, **h**, **j**, **l**, **m**). *P < 0.05, **P < 0.01, ***P < 0.001; ns no significance

We then investigated whether NELL2 signals through Fn1/ITGB1. The focal adhesion and PI3K-Akt pathways are known to regulate osteoblast and adipocyte differentiation.^29,30^ Thus, we examined the impact of NELL2 and Fn1 on the FAK/AKT pathway. As expected, the levels of pFAK (Y397) and pAKT (S473) were upregulated following overexpression of NELL2 (Fig. 6a, b). Conversely, these protein levels were downregulated in NELL2 knockout cells or in ST2 cells following knockdown of Fn1 (Fig. S14a–d). Furthermore, when NELL2 was overexpressed in the context of Fn1 knockdown, there was no increase in the levels of pFAK (Y397) and pAKT (S473) (Fig. 6c, d). Moreover, in ST2 cells that overexpressed NELL2, co-transfection with Fn1 C-terminal construct, treatment with RGD peptide, or co-transfection with ITGB1 siRNA, effectively prevented the activation of pFAK (Y397) and pAKT (S473) induced by NELL2 (Fig. 6e–h; Fig. S14e, f). These results indicate that NELL2 regulates the FAK-AKT pathway through Fn1/integrins.

We subsequently treated ST2 cells with Defactinib, a specific FAK inhibitor. The results demonstrated that Defactinib significantly reduced pFAK (Y397) and pAKT (S473) (Fig. 6i, j). Furthermore, Defactinib impaired osteoblast differentiation while promoting adipocyte differentiation, and largely mitigated both the pro-osteogenic and anti-adipogenic effects of NELL2 (Fig. 6k–m; Fig. S15a–c; Fig. S16a–f). These findings underscore the crucial role of the FAK/AKT pathway in mediating the effects of NELL2 on osteoblast and adipocyte differentiation.

### Administration of NELL2-AAV ameliorated bone loss in OVX mice

**Fig. 7:**
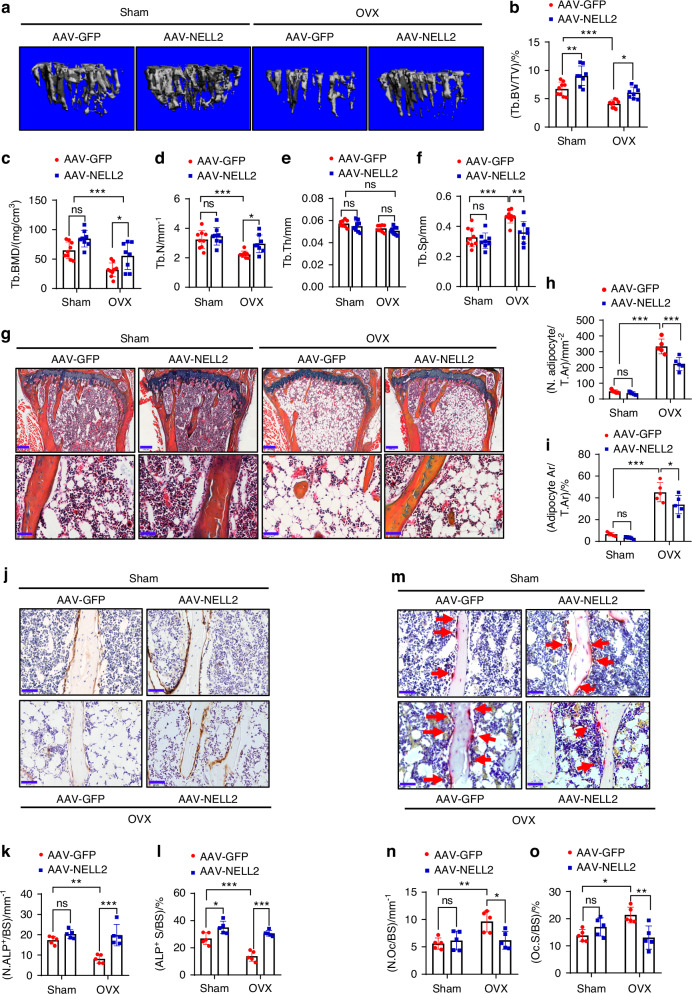
Administration of NELL2-AAV ameliorated bone loss in OVX mice. **a** Cancellous bone mass at proximal tibial metaphysis in the mice was analyzed using μCT, and the representative reconstruction images are shown. **b**–**f** Histomorphometric parameters of cancellous bone were quantified, *n* = 8–9. **g** ABH/OG staining was conducted. Scale bar: 200 μm (upper panel); 50 μm (lower panel). **h**, **i** The numbers and areas of adipocytes were quantified, *n* = 5. **j** Representative images of ALP IHC staining are shown. **k**, **l** The numbers and surface percentage of ALP^+^ osteoblasts were quantified, *n* = 5. **m** Representative images of TRAP staining are shown. **n**, **o** The numbers and surface percentage of osteoclasts were quantified, *n* = 5. Scale in (**j**, **m**): 50 μm. Data are mean ± SD. Comparisons were conducted using two-way ANOVA followed by Tukey’s test, *P < 0.05, **P < 0.01, ***P < 0.001; ns no significance

To determine the potential of NELL2 to enhance bone health, we generated an adeno-associated virus (AAV) expressing NELL2 (NELL2-AAV) and delivered it via tail vein injection to mice. The tissue distribution of NELL2-AAV was examined using fluorescence microscopy one month after injection. The results demonstrated effective targeting of NELL2-AAV to bone, as well as cardiac muscle, skeletal muscle, and kidney (Fig. S17a). qRT-PCR analysis confirmed that NELL2 was overexpressed in both tibial bone and isolated BMSCs (Fig. S17b, c). Subsequently, we established the OVX mouse model, and the mice were systemically administered with AAV carrying either GFP or NELL2, respectively. As expected, after 2 months, OVX/AAV-GFP mice exhibited diminished bone mass compared with Sham/AAV-GFP mice. Specifically, Tb. BV/TV, Tb. BMD and Tb. N were decreased by 40.4%, 51.0% and 31.2%, respectively, while Tb. Sp was increased by 43.8% at the tibial metaphysis (Fig. 7a–f). Additionally, cortical thickness was reduced by 10.5% in OVX/AAV-GFP mice versus Sham/AAV-GFP mice (Fig. S18a, b). After administration of NELL2-AAV, Tb. BV/TV was significantly increased in Sham/AAV-NELL2 group compared to Sham/AAV-GFP group, while Tb. BMD and Tb. N exhibited a rising tendency although they did not attain statistical significance (Fig. 7a–f). Notably, the upregulation of NELL2 in OVX/AAV-NELL2 mice significantly mitigated cancellous bone loss induced by OVX, as indicated by increased Tb. BV/TV (50.0%), Tb. BMD (74.7%) and Tb. N (31.8%), along with decreased Tb. Sp (20.0%) compared with OVX/AAV-GFP mice (Fig. 7a–f).

Compared to Sham/AAV-GFP mice, OVX/AAV-GFP mice demonstrated an increase in the number and area percentage of marrow adipocytes (Fig. 7g–i), as well as a decrease in the number and surface percentage of ALP-positive osteoblasts, and an increase in the number and surface percentage of TRAP^+^ osteoclasts (Fig. 7j–o). In contrast, when compared to OVX/AAV-GFP mice, the upregulation of NELL2 in OVX/AAV-NELL2 mice resulted in a reduction in the number and area percentage of marrow adipocytes, an increase in the number and surface percentage of ALP-positive osteoblasts (Fig. 7g–l), and a decrease in the number and surface percentage of TRAP-positive osteoclasts (Fig. 7m–o). In addition, the upregulation of NELL2 in Sham/AAV-NELL2 mice increased the surface percentage of ALP-positive osteoblasts compared with Sham/AAV-GFP (Fig. 7j, l). Together, these results suggest that NELL2 is beneficial to the prevention of osteoporosis.

## Discussion

NELL1 and NELL2 are the two mammalian homologs of chicken Nel. NELL1 was initially found to be upregulated in patients with craniosynostosis.^31,32^ NELL1 plays important roles in bone development and regeneration.^33,34^ While excessive NELL1 led to premature cranial suture fusion and skeletal overgrowth in mice,^35^ NELL1 deficiency resulted in perinatal lethality and skeletal abnormalities in skull and vertebral column due to impaired intramembranous and endochondral ossification.^26,36,37^ Additionally, haploinsufficiency of NELL1 in mice led to age-related osteoporosis associated with impaired osteoblastic and excessive osteoclastic activity.^24^ Although there is a structural similarity between NELL1 and NELL2, the role of NELL2 in bone remains unexplored.

As a secreted protein, NELL2 has been detected in the cerebrospinal fluid of humans, originating from neurons and serving as a neurotransmitter.^38^ In this study, we observed a decrease in serum levels of NELL2 in postmenopausal individuals with osteopenia and osteoporosis compared to those with normal BMD. Although the exact source of serum NELL2 remains unclear at present, we found that NELL2 protein is predominantly expressed in the heart, brown adipose tissue, bone, kidney, muscle and brain in mice. Interestingly, the expression profiles of NELL2 in the form of mRNA and protein exhibit notable differences. This discrepancy may be attributed to post-transcriptional regulatory mechanisms such as mRNA stability, translation efficiency, or protein degradation that affect NELL2 protein levels in different tissues.^39^

Previous studies have focused on the function of NELL2 in the nervous system, where it regulates neural cell proliferation and differentiation, axon guidance, spatial learning, and feeding-related behaviors.^19,20,22,40–42^ The function of NELL2 in bone remains unknown. Our study revealed that NELL2 protein expression was decreased in bone of aged and OVX mice. These data suggest that the decrease of NELL2 in bone is associated with the development of osteoporosis and that the serum levels of NELL2 may reflect bone mass quantity. Furthermore, NELL2 protein increased during osteogenic differentiation in stromal cells, suggesting its involvement in this process. As expected, we demonstrated NELL2 promoted osteoblast differentiation and suppressed adipocyte differentiation. Collectively, these findings indicate that NELL2 favors osteoblast differentiation over adipocyte formation from stromal progenitor cells.

To determine the physiological role of NELL2 in bone, we generated mice with deletion of NELL2 specifically in preosteoblasts. A significant reduction in cancellous bone mass was observed in the mutant mice. Histological analysis further revealed a decrease in both osteoblast and osteoclast numbers. The dynamic histomorphometric parameters reflecting osteoblast activity were decreased and serum levels of PINP and CTX-1 were diminished, indicating a reduced bone turnover rate in the mutant mice. It is thus inferred that the bone loss phenotype observed in the NELL2 deficient mice primarily stems from a more pronounced decrease in bone formation than in bone resorption, ultimately culminating in an overall decline of bone mass. Moreover, knockout of NELL2 in preosteoblasts does not alter the expression level of NELL1, suggesting that these two proteins function independently in bone homeostasis.

In line with the decrease in bone formation, calvarial preosteoblasts from NELL2 knockout mice exhibited diminished osteogenic potential and enhanced adipogenic potential. Regarding osteoclastogenesis, our findings indicate that deletion of NELL2 reduced Rankl/opg ratio in preosteoblasts and impaired the capacity of osteoclast precursor cells to develop into osteoclasts, implying an indirect mechanism by which NELL2 influences osteoclast differentiation. Collectively, our findings demonstrate that NELL2 expressed in preosteoblasts promotes both osteoblast and osteoclast differentiation. However, the promotion of osteoblast differentiation predominates over osteoclast differentiation. Consequently, the overall impact of NELL2 tends to increase bone mass.

We then endeavored to decipher how NELL2 regulates osteoblast differentiation. ROBO3 and ROS1 are the two known receptors for NELL2, both possessing fibronectin type III (FNIII) domains through which they interact with NELL2.^22,23^ Slit/ROBO signaling pathway plays a role in bone homeostasis.^43^ Amongst the members of the ROBO family, ROBO3 is expressed in osteoclastic lineages rather than osteoblastic lineages.^43^ Regarding the ROS1 receptor, its knockdown in BMSCs resulted in a diminished potential for osteogenic differentiation.^44^ However, based on our RNA-seq data, the expression of ROS1 in preosteoblasts in mice is negligible. Therefore, we conducted LC-MS/MS to identify potential receptors or binding partners of NELL2 on the membrane of BMSCs and discovered an interaction between NELL2 and Fn1.

Despite the absence of signaling and transmembrane domains, Fn1 plays a crucial role in cell signaling by binding various factors to regulate their distribution, activation, and presentation to cells.^45^ Notable examples include vascular endothelial growth factor (VEGF), hepatocyte growth factor (HGF) and transforming growth factor-beta (TGF-β), which interact with Fn1, forming complexes that integrate signaling along with their cognate receptors.^46–49^ Herein, we demonstrated that NELL2 specifically bound to the C-terminal FNI domain of Fn1, rather than the FNIII domain where the RGD motif is located. This binding pattern is similar to Pref-1, a transmembrane protein containing EGF repeats. Pref-1 undergoes proteolytic cleavage, resulting in a biologically active soluble form, which activates integrin-mediated downstream signaling through its interaction with the C-terminus of Fn1, thereby inhibiting adipocyte differentiation.^50^ Intriguingly, our domain-specific competitive inhibition experiments revealed that Fn1 C-terminal-HA fragment disrupted the interaction between NELL2 and full-length Fn1, and largely mitigated the pro-osteogenic and anti-adipogenic effects of NELL2. These results indicate that NELL2 regulates osteoblast and adipocyte differentiation through interacting with Fn1.

As a key extracellular matrix component, Fn1 is implicated in essential physiological processes such as cell adhesion, migration, and cell fate determination.^51,52^ Fn1 exists in two forms: plasma Fn1 and cellular Fn1. Plasma Fn1, which lacks extra domain A (EDA) and extra domain B (EDB), is synthesized by hepatocytes and circulates in an inactive conformation. Cellular Fn1, on the other hand, is synthesized by various cell types including fibroblasts and osteoblasts. It contains EDA and EDB domains and contributes to the assembly of the extracellular matrix.^53^ While deletion of liver-derived circulating Fn1 led to reduced bone matrix mineralization in mice, deletion of osteoblast-derived Fn1 affected osteoblast function.^54^ Further research demonstrated the role of Fn1 in osteoblast and adipocyte differentiation via distinct integrins.^50,55^

Our RNA-seq analysis unveiled that both NELL2 and Fn1 regulated actin cytoskeleton, focal adhesion, PI3K-Akt, and cell adhesion pathways that are primarily associated with integrins. Integrins are a family of heterodimeric transmembrane proteins composed of α and β subunits.^56^ Osteoblasts and osteoclasts express different types of integrins.^57^ We found that treating ST2 cells with RGD peptide or silencing ITGB1 resulted in decreased osteogenesis and enhanced adipogenesis. Moreover, the impact of NELL2 on osteoblast and adipocyte differentiation was largely mitigated in RGD peptide-treated cells or in ITGB1-silenced cells, indicating that Fn1/integrin interaction is required for the role of NELL2. Of equal importance, Fn1 is required for the interaction between NELL2 and ITGB1 since knockdown of Fn1 reduced the interaction. Collectively, the data suggest that NELL2 may regulate osteoblast and adipocyte differentiation through forming a complex with Fn1 and integrin.

Upon fibronectin-integrin binding, FAK is recruited and phosphorylated, initiating the activation of crucial signaling proteins such as PI3K, mitogen-activated protein kinases (MAPKs), protein kinase C (PKC), and Rho family GTPases.^58^ Previous studies have shown that mice lacking FAK in mesenchymal progenitor cells displayed reduced osteoblasts, low bone mass, increased bone marrow adiposity, along with inactivated AKT/mTOR signaling.^59,60^ These indicate that FAK/AKT signaling is required for osteoblast differentiation. NELL1 was previously demonstrated to promote osteoblast differentiation through integrins-mediated activation of FAK and ERK1/2.^61^ In our study, NELL2 overexpression increased the levels of pFAK (Y397) and pAKT (S473), which was attenuated by blocking NELL2/Fn1 interaction or Fn1/integrin interaction, or knocking down NELL2, Fn1 or ITGB1 expression, suggesting that NELL2 activates FAK/AKT signaling pathway through NELL2/Fn1/integrin complex. Furthermore, Defactinib inactivated FAK/AKT signaling and mitigated the pro-osteogenic and anti-adipogenic effects of NELL2, indicating that NELL2 regulates osteoblast and adipocyte differentiation via the FAK/AKT pathway.

Thus far our utmost interest is to determine whether NELL2 possesses the potential to benefit bone health. rAAV9 has been demonstrated to effectively transduce osteoblastic lineage cells in bone.^62^ In this study, we generated a rAAV9 to overexpress NELL2. Notably, the systemic administration of NELL2-AAV effectively targeted bone and ameliorated bone loss induced by OVX. In addition, NELL2-AAV also increased cancellous bone mass in Sham/NELL2-AAV mice, as evidenced by an increase in Tb. BV/TV. The underlying cellular basis involved an increase in osteoblasts and a decrease in marrow adipocytes. Of interest, the findings that NELL2-AAV reduced osteoclast numbers in OVX mice seems to contradict the decrease in osteoclasts in NELL2 deficient mice. Although not fully understood, this discrepancy may be attributed to the nonspecificity of NELL2-AAV in transducing various cells compared to the specific deletion of NELL2 in the conditional deficient mice. Further research is needed to determine if NELL2 expressed in osteoclasts affects osteoclastogenesis differently than in osteoblasts.

In summary, the findings of this study have established NELL2 as a crucial regulator of bone homeostasis. The expression of NELL2 in preosteoblastic cells plays a significant role in promoting osteoblast differentiation and bone formation, while simultaneously inhibiting marrow adipocyte formation. These effects are mediated through its interaction with fibronectin 1 to activate the integrin-mediated FAK/AKT signaling pathway (Fig. S19). Moreover, the study indicates that enhancing NELL2 levels therapeutically may hold promise for ameliorating osteoporosis.

## Materials and methods

### Animals

Nell2 floxed mice were gifted by Professor Alexander Jaworski at Brown University. Preosteoblast-specific Nell2 knockout mice (Col1-cre;*Nell2*^fx/fx^) were generated by crossing Nell2 floxed mice with Col1-cre mice (driven by the 2.3 kb mouse Col1a1 promoter). Genomic DNA was extracted from tail tips and genotyping PCR was performed using a kit by Vazyme (Nanjing, China). The primers for genotyping and those for validating NELL2 knockout efficiency are listed in Table S3.

For OVX model and AAV delivery, three-month-old female C57BL/6 mice were purchased from Huafukang Bioscience (Beijing, China) and underwent either ovariectomy (OVX) or sham operation under anesthesia. To produce AAV-NELL2, the NELL2 CDS sequence was cloned into pcAAV-CMV-P2A-GdGreen-tWPA and packaged with AAV9 capsids (OBiO Tech, Shanghai, China). For systemic delivery of AAV, 100 μL (4×10^11^ vg) of AAV-NELL2 or AAV-GFP was injected into the mice via tail vein 3 days after surgery. Tissues were collected one month after injection to evaluate NELL2 overexpression. Tibiae were collected two months after injection for μCT analysis and histological staining.

All animal studies were approved by the Animal Ethical and Welfare Committee of Tianjin Medical University Chu Hsien-I Memorial Hospital (DXBYY-IACUC-2022008). The sample size was determined based on a survey of published literatures. Mice were housed in a standard individually ventilated cage system (Tecniplast, Italy) in a specific pathogen-free facility, with each cage accommodating five mice. The housing environment maintained a 12-h light/dark cycle, with controlled temperature (22 °C) and humidity levels (50%–60%). Mice were provided with autoclaved food and water ad libitum.

### Immunofluorescence

Dissected bones were fixed in 4% paraformaldehyde overnight, decalcified in 10% EDTA for 4 days and dehydrated in 30% sucrose for 2 days. Soft tissues were fixed, followed by sucrose dehydration. All the samples were then embedded in OCT compound and sectioned for immunofluorescence analysis.

### μCT analysis

Tibiae were collected, fixed in 4% paraformaldehyde for 3 days, and subjected to μCT scanning and analysis using the VivaCT 80 system (SCANCO Medical AG, Switzerland). The scanner parameters were set at an energy of 55 kVp, an intensity of 145 μA, an integration time of 300 ms, and a voxel size of 10.4 μm. The region of interest (ROI) for cancellous bone was defined as a segment starting 100 μm beneath the growth plate and extending 1 mm in length. In contrast, the ROI for cortical bone starts 2 mm below the growth plate and is 0.5 mm long.

### Calcein double labeling

The mice were intraperitoneally injected with calcein (10 mg/kg) 7 days and 2 days prior to euthanasia. Femurs were isolated and fixed in 4% paraformaldehyde. After dehydration, the samples were embedded in Technovite 9100 (Kulzer, German). Non-decalcified bone sections (8 μm) were cut, and analyzed using OsteoMeasure system (OsteoMetrics, Atlanta, GA, USA) under fluorescence microscope to determine dynamic bone histomorphometric parameters such as MAR and BFR/BS.

### ABH/OG staining and TRAP staining

Tibiae were harvested, fixed in 10% formalin for 3 days, and decalcified in 14% EDTA for 14 days, followed by embedding in paraffin. Longitudinally oriented bone sections with a thickness of 5 μm were subjected to standard ABH/OG staining or TRAP staining. The ROI was selected as a 1 mm segment, beginning 100 μm beneath the growth plate. The number and area percentage of adipocytes, as well as the number and surface percentage of TRAP-positive osteoclasts, were determined.

### IHC staining

Bone sections were digested with 0.05% trypsin at 37 °C for 20 min for antigen retrieval, and then incubated with primary antibody against osteocalcin (Proteintech, Wuhan, China) or ALP (HuaBio, Hangzhou, China) at 4 °C overnight. Subsequently, an HRP-DAB system (Proteintech, Wuhan, China) was used to detect the immunoactivity, followed by counterstaining with hematoxylin.

### Human serum samples collection and BMD measurements

Human serum samples were obtained from patients who participated in a previously approved institutional study (ZXYJNYYsMEC2023-12). All individuals provided written informed consent to have their samples stored for future use. The human study was approved by the Medical Ethics Committee of Tianjin Medical University Chu Hsien-I Memorial Hospital. The study included 28 postmenopausal women who had no history of hormone use, or anti-osteoporosis treatment. BMD of the femur neck, total hip, and lumbar spine (L1-4) was measured using dual-energy X-ray absorptiometry (DXA) (GE Healthcare, Chicago, IL, USA). T-scores were derived from the BMD values for each skeletal site. The participants were classified into three groups based on the scores: normal bone mass (T-score ≥−1.0), osteopenia (−1.0 > T-score > −2.5), and osteoporosis (T-score ≤−2.5) at any of the three sites. Peripheral blood samples were collected, and the serum was harvested and stored at −80 °C for further analysis.

### ELISA assays

Serum concentrations of PINP and CTX-1 in mice were measured using the respective PINP and CTX-1 ELISA Kits (Signalway Antibody Biotech, USA). Serum levels of human NELL2 in postmenopausal women were determined using a human NELL2 ELISA Kit (Signalway Antibody Biotech, USA). All measurements were conducted according to manufacturer’s instructions.

### Cell culture

ST2 cells were maintained in α-MEM containing 10% FBS. Primary BMSCs were collected from femurs and tibiae of mice and grown in α-MEM containing 10% FBS in a 10-cm dish. Primary preosteoblasts were collected from the calvaria of 3-day-old mice by sequential trypsin/collagenase digestions, and cultured in α-MEM medium supplemented with 10% FBS. For adipocyte differentiation, the cells reaching 100% confluence were treated with adipogenic medium (α-MEM containing 10% FBS, 0.5 μmol/L dexamethasone, 0.25 mmol/L methylisobutylxanthine, 5 μg/mL insulin, and 50 μmol/L indomethacin) for 3 days, followed by treatment with 5 μg/mL insulin alone for additional 2-3 days. For osteoblast differentiation, the cells reaching 80% confluence were cultured in osteogenic medium (α-MEM containing 10% FBS, 50 μg/mL ascorbic acid, and 5 mmol/L β-glycerophosphate) for 14–21 days.

### Constructs and transfection

The NELL2-Myc expression construct was purchased from Origene (Rockville, MD, USA). Sequential deletions were conducted to generate a series of NELL2 truncates using Mut Express® II Fast Mutagenesis Kit (Vazyme, Nanjing, China) with the wild-type expression construct as a template. Full-length of Fn1 CDS construct, and Fn1 deletion mutants were generated by using pEASY-Basic Seamless Cloning and Assembly Kit (TransGen, Beijing, China). Fn1 C-terminal-HA construct was generated by inserting sequence of the Fn1 fragment (aa 2 296-2 425 domain) into pcDNA3.1^+^ vector in frame with the N-terminal signal sequence in the vector using pEASY-Basic Seamless Cloning and Assembly Kit (TransGen, Beijing, China). All the primers are listed in Table S4.

For NELL2 gain-of-function study, ST2 cells were transfected with the NELL2-Myc expression plasmid or the vector for 16 h using lipofectamine 3000 reagent (Invitrogen, Carlsbad, CA, USA). For NELL2 or Fn1 loss-of-function studies, ST2 cells were transfected with 30 nmol/L of NELL2 siRNAs, Fn1 siRNAs, or control siRNA (Genepharma, Shanghai, China) using lipofectamine RNAimax (Invitrogen, Carlsbad, CA, USA). For co-transfection studies, ST2 cells were transfected with NELL2-Myc expression construct (or vector) and 30 nmol/L Fn1 siRNAs (or control siRNA) or 30 nmol/L ITGB1 siRNA (or control siRNA) or Fn1 C-terminal construct (or control vector) using lipofectamine 3000. Subsequently, adipogenic or osteogenic induction was performed to allow the cells to differentiate.

### RNA extraction and qRT-PCR

For tissue samples, RNA was extracted using RNAiso Plus (Takara, Japan). For cells, RNA was extracted using a total RNA isolation kit (Omega Bio-Tek, Norcross, GA, USA). 500 ng RNA was reverse transcribed into cDNA with PrimeScript™ RT Master Mix (Takara, Japan). Subsequently, cDNA was PCR-amplified with a SYBR Green fluorescence PCR kit (ABclonal Technology, Wuhan, China). All the PCR primers used are listed in Table S5. The relative expression level of each target gene was normalized against β-actin and determined as 2^-ΔΔCT^.

### RNA-seq

Total RNA was isolated using a total RNA isolation kit (Omega Bio-Tek, Norcross, GA, USA), and mRNA is enriched by oligo (dT)-attached magnetic beads. Sequencing libraries were generated using Hieff NGS® MaxUpTM II Dual-model mRNA Library Prep Kit for Illumina (YEASEN, Shanghai, China). The sequencing library was then sequenced on a MGISEQ-2000 platform (BGI, Shenzhen, China) and 150 bp paired-end reads were generated. Reads from each library were mapped to mouse transcriptome and filtered using HISAT2 software, following Strand alignment and filtering pipelines. Reads were normalized using DESeq. Fold change and *P* value was calculated using moderated 2-tailed *t* test. The data were used for bioinformatics analysis using the Dr. Tom Biosystem (BGI, Shenzhen, China).

### Pull down assay and mass spectrometry analysis

Membrane proteins from BMSCs were extracted using a Membrane and Cytosol Protein Extraction Kit (Beyotime, Shanghai, China). For pull down assay, the isolated membrane proteins were incubated with His-labeled recombinant NELL2 (R&D Systems, MN, USA) and anti-His antibody (Abmart, Shanghai, China), followed by adsorption to protein A/G magnetic beads (MedChemExpress, NJ, USA) at room temperature for 30 min. The magnetic beads were washed, magnetically separated and eluted. The eluted proteins were separated by SDS-PAGE, and the bands of interest were excised from the SDS-PAGE gel, decolorized, alkylated, and digested with trypsin. The resulting peptides were extracted, desalted, and analyzed with a nano-UPLC (EASY-nLC1200) coupled to a Q Exactive HFX Orbitrap instrument (Thermo Fisher Scientific, MA, USA) with a nanoelectrospray ion source. Data dependent acquisition (DDA) was performed with Orbitrap analyzer in positive mode, and the top 10 most intense ions were fragmented by Higher-energy Collisional Dissociation (HCD). Vendor’s raw MS files were processed using Proteome Discoverer (PD) software (Version 2.4.0.305). MS spectra lists were searched against their species­level UniProt FASTA databases, carbamidomethyl (C) as a fixed modification, oxidation (M) and acetyl (protein N­term) as variable modifications. A maximum of 2 missed cleavage(s) was allowed. The false discovery rate (FDR) was set to 0.01 for both peptide-spectrum match (PSM) and peptide levels. Peptide identification was performed with an initial precursor mass deviation of up to 10 × 10^−6^ and a fragment mass deviation of 0.02 Da.

### Co-Immunoprecipitation (Co-IP)

The cells transfected with NELL2-Myc and Fn1-HA expression constructs were lysed and the cell lysates were collected and subjected to immunoprecipitation using protein A/G magnetic beads conjugated with an antibody against HA-Tag (Affinity Biosciences, OH, USA) or Myc-Tag (ABclonal, Wuhan, China), respectively. Following incubation, the beads were washed to eliminate nonspecifically bound proteins, and the immunoprecipitated complex was eluted, and subsequently subjected to western blotting using anti-Myc or anti-HA antibody.

### Western blot

Total protein was extracted using RIPA Lysis Buffer (Sangon Biotech, Shanghai, China) and protein concentration was determined by BCA Protein Quantification Kit (Vazyme, Nanjing, China). Then the proteins were separated by SDS-PAGE and transferred onto PVDF membranes. After blocking with 5% milk for 1 h, the membranes were incubated overnight with primary antibodies (Table S6). This was followed by incubation with horseradish peroxidase (HRP)-conjugated IgG for 1 h. Finally, chemiluminescence reagent (Advansta, San Jose, USA) was used to visualize the results.

### ALP staining and alizarin red S staining

For ALP staining, the cells that underwent 14 days of osteogenic induction were fixed in 4% paraformaldehyde for 10 min and subsequently stained with BCIP/NBT staining solution (Beyotime Biotech, Shanghai, China) for 15 min. For alizarin red S staining, the cells following 21 days of osteogenic induction were fixed, and then stained with 1% alizarin red S (pH 4.2) for 5 min. To quantify the staining intensity, the dye was extracted using 10% acetic acid and the absorbance of the extracted dye was measured at 405 nm.

### ALP activity measurement

The cells were lysed and ALP activity was measured using an assay kit (Beyotime, Shanghai, China) by reading the optical density of the cell lysates at 405 nm. Total protein content was quantified using the BCA protein quantification kit by reading the optical density at 562 nm. The enzymatic activity of ALP was normalized to the total protein content of each sample by calculating the ratio between the OD values.

### Oil red O staining

Fully differentiated adipocytes were gently washed twice with PBS and fixed in 4% paraformaldehyde for 10 min. The samples were washed twice with deionized water and stained in 60% saturated oil red O solution for 5 min. For quantification, the oil red O dye was extracted using isopropanol, and its absorbance at 520 nm was measured.

### Osteoclast differentiation assay

Bone marrow cells were flushed from the femurs and tibiae of mice, and seeded in DMEM ( ∼ 4 × 10^6^/mL) supplemented with 10% FBS and 20 ng/mL macrophage colony-stimulating factor (M-CSF). After 3 days, the cells continued to be cultured in presence of 20 ng/mL M-CSF and 10^−8^ mol/L 1,25(OH)_2_D_3_ to induce osteoclast differentiation. The culture medium was refreshed every 3 days. After 6–8 days of induction, the cells were subjected to TRAP staining using a kit (Sigma-Aldrich, USA). TRAP-positive osteoclasts with nuclei≥3 were counted.

### Statistical analysis

Data are expressed as the mean ± SD. For relative mRNA and protein quantification, the means of the control groups are set to 1. Student’s *t* test was used for two-group comparisons. One-way analysis of variance (ANOVA) was conducted for multiple group comparisons with one independent variable, followed by Dunnett’s test for post-hoc comparisons. Two-way ANOVA was employed for multiple group comparisons with two independent variables, followed by Tukey’s test.

## Supplementary information

Supplementary Figures and Tables

## Data Availability

The RNA-seq data generated in this study are available at the NCBI Gene Expression Omnibus repository (GSE266198 and GSE265936). The data and materials that support the findings of this study are available from the corresponding author upon request.
