# Supplementary material for: NELL2, a novel osteoinductive factor, regulates osteoblast differentiation and bone homeostasis through fibronectin 1/integrin-mediated FAK/AKT signaling
Source: Bone Res. 2025 Apr 11;13:46. doi: 10.1038/s41413-025-00420-5 (PMC11986068; doi:10.1038/s41413-025-00420-5)
Supplement: Supplementary file 1 — Supplementary Figures and Tables [file 41413_2025_420_MOESM1_ESM.docx]

**Supplementary information for:**

**NELL2, a novel osteoinductive factor, regulates osteoblast differentiation and bone homeostasis through fibronectin 1/integrin-mediated FAK/AKT signaling**

Hairui Yuan*, Xinyu Wang, Shuanglin Du, Mengyue Li, Endong Zhu, Jie Zhou, Yuan Dong, Shuang Wang, Liying Shan, Qian Liu, Baoli Wang*

NHC Key Lab of Hormones and Development, Tianjin Key Lab of Metabolic Diseases, Chu Hsien-I Memorial Hospital & Institute of Endocrinology, Tianjin Medical University, Tianjin 300134, China

***Correspondence to:**

Baoli Wang, E-mail: blwang@tmu.edu.cn; Hairui Yuan, E-mail: yuanhairui@tmu.edu.cn

**This file includes:**

Supplementary Figure S1-S19

Supplementary Table S1-S6

**
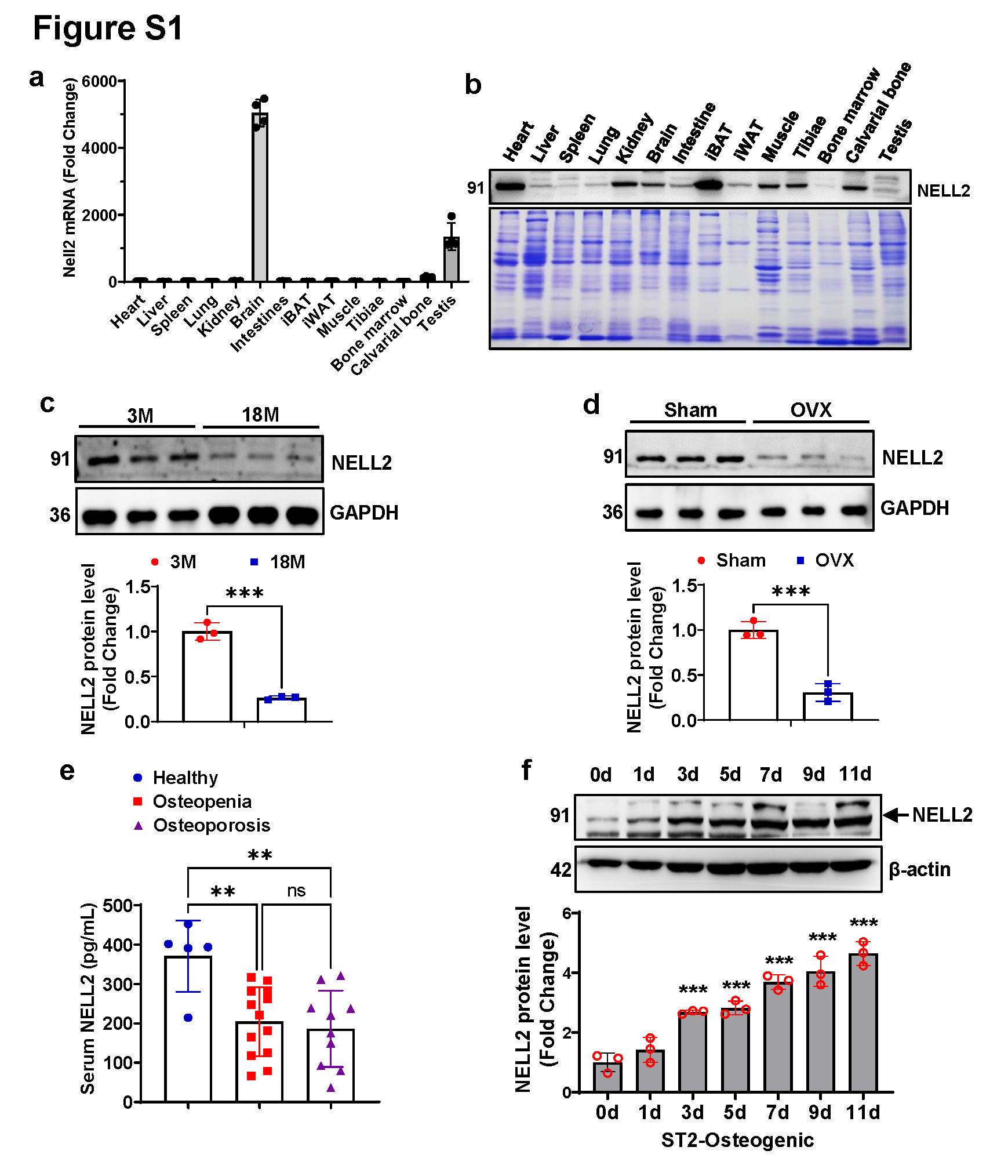
**

**Figure S1. NELL2 was diminished in the bone of aged and OVX mice and in the serum of osteoporosis patients.**

(a) qRT-PCR was performed to detect the mRNA expression of NELL2 in various mouse tissues (n=4). (b) Western blotting was performed to detect NELL2 protein levels in various mouse tissues (20 μg protein per sample), with Coomassie Brilliant Blue-stained total protein serving as an internal reference. (c) NELL2 protein levels in the tibiae were compared between 18-month-old aged and 3-month-old young mice, n=3. (d) NELL2 protein levels in the tibiae were compared between OVX and Sham mice, n=3. (e) Serum NELL2 levels were measured in postmenopausal healthy individuals and osteopenia or osteoporosis patients, Healthy: n=5, Osteopenia: n=13, Osteoporosis: n=10. (f) NELL2 protein levels were detected during osteogenic differentiation in ST2 cells, n=3. Data are mean ± SD. Comparisons were conducted using Student’s t test (c, d), or one-way ANOVA followed by Tukey’s test (e), or followed by Dunnett’s test (f), **p<0.01, ***p<0.001; ns: no significance

**
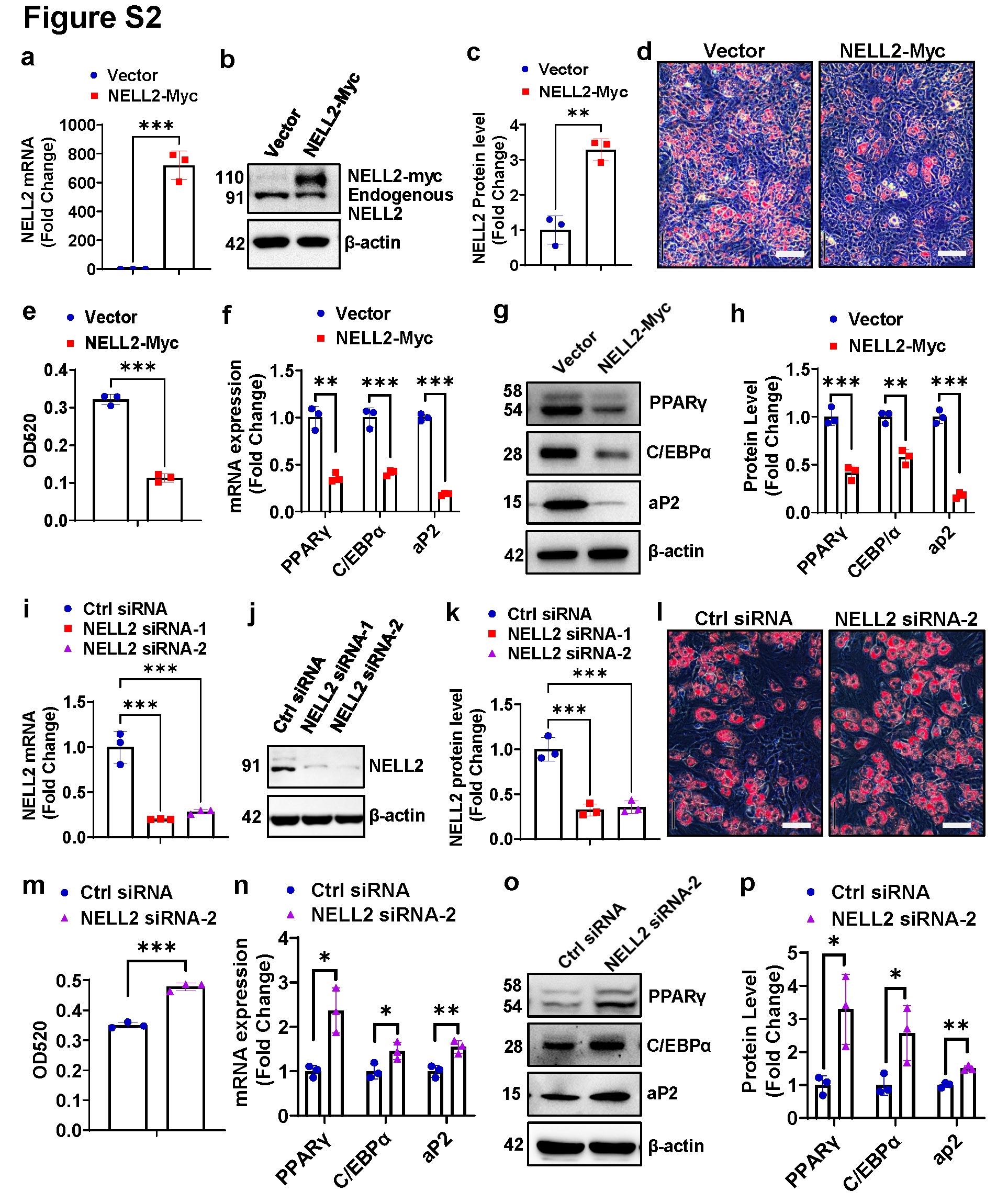
**

**Figure S2. NELL2 inhibited adipocyte differentiation.**

ST2 cells were cultured and induced to allow adipogenic differentiation after overexpression (a-h) or knockdown (i-p) of NELL2. The overexpression (a-c) or knockdown (i-k) of NELL2 was verified by qRT-PCR (a, i) or western blotting analysis (b and c, j and k). (d, l) Differentiated adipocytes were stained with oil red O after 5-6 days of induction. (e, m) Oil red O extracted with isopropanol was measured at OD520. (f-h, n-p) The mRNA (f, n) and protein levels (g and h, o and p) of adipogenic factors were examined. Scale in (d, l): 100 μm. Data are mean ± SD, n=3, Comparisons were conducted using Student’s t test (a, c, e, f, h, m, n, p), or one-way ANOVA followed by Dunnett’s test (i, k), *p<0.05, **p<0.01, ***p<0.001.


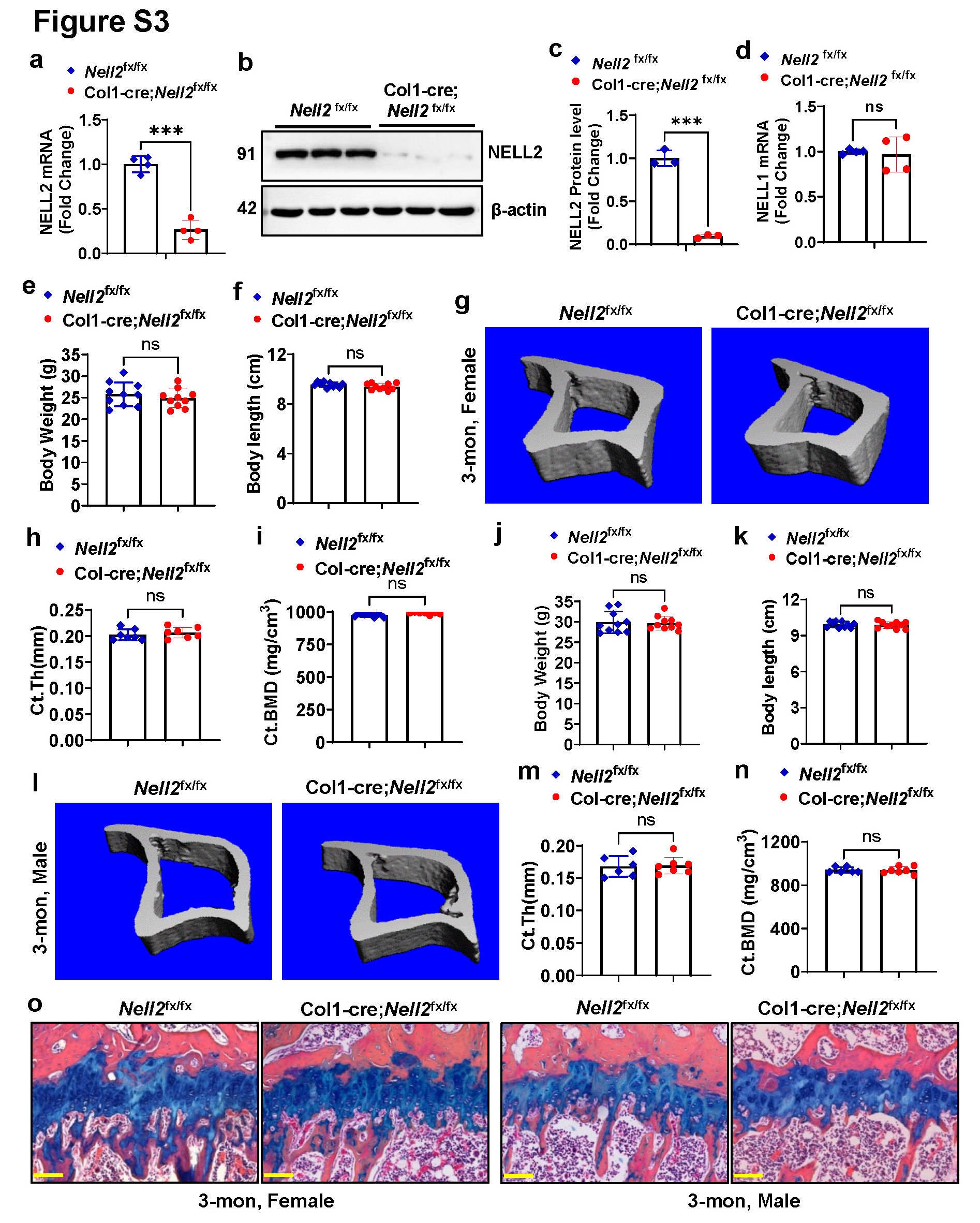


**Figure S3. Preosteoblast-specific deletion of NELL2 did not alter body length, body weight, cortical bone mass and growth plate morphology.**

(a-c) qRT-PCR (a, n=4) and western blotting (b, c, n=3) was performed to confirm the knockout of NELL2 in calvarial preosteoblasts. (d) The expression of NELL1 in NELL2-deficient calvarial preosteoblasts was analyzed by qRT-PCR. (e and f, j and k) Body weight (e: Female, j: Male) and body length (f: Female, k: Male) were measured in 3-month-old mice, n=10. (g-i, l-n) Histomorphometric parameters for cortical bone were analyzed using μCT (g-i: Female, n=7; l-n: Male, n=6-7). Data are mean ± SD. (o) Representative images of growth plate morphology are shown (left panel: Female; right panel: Male). Scale bar: 100 μm. Comparisons were conducted using Student’s t test, ***p<0.001; ns: no significance.

**
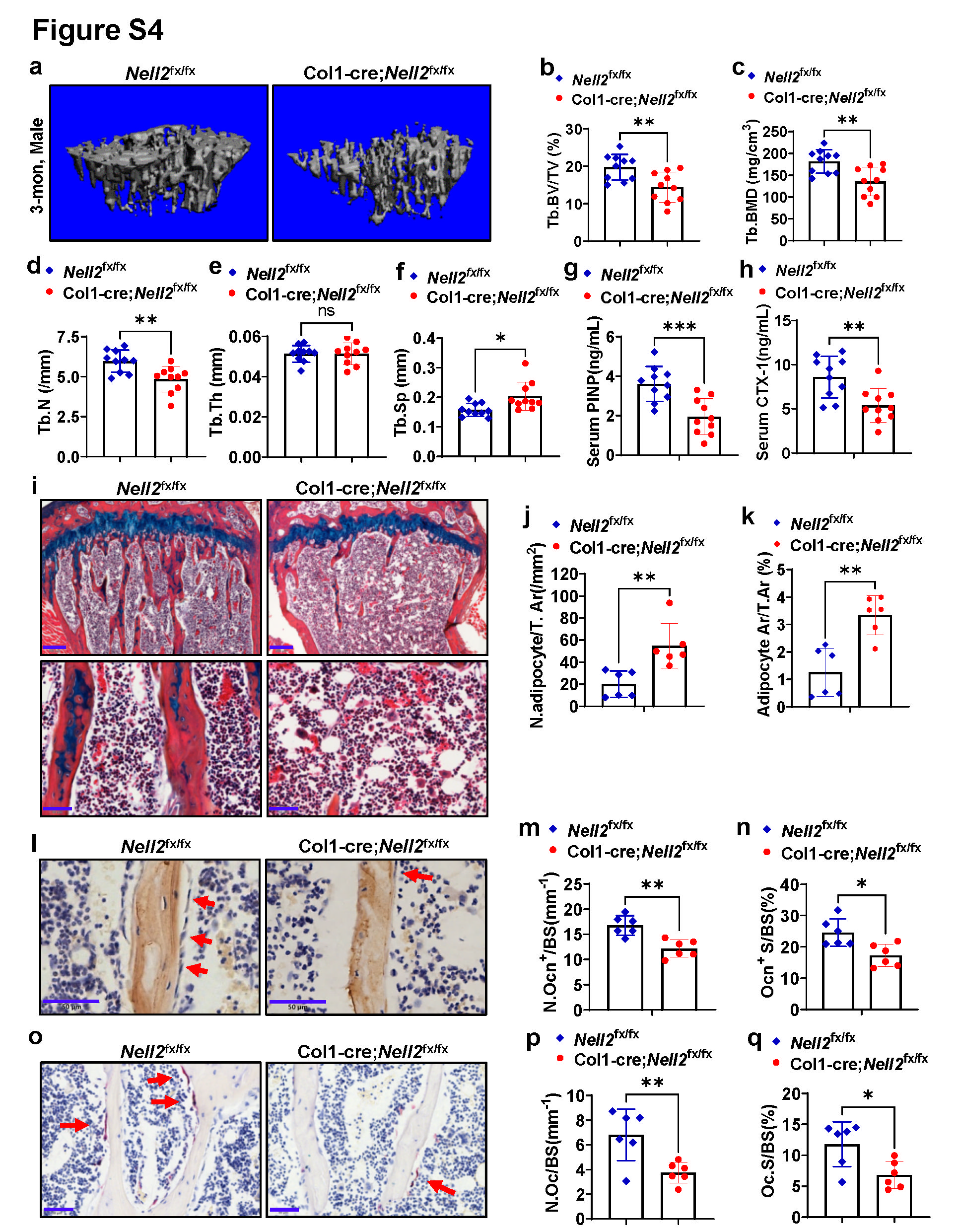
**

**Figure S4. Preosteoblast-specific deletion of NELL2 reduced bone mass in male mice.**

(a-f) Cancellous bone mass at proximal tibial metaphysis in 3-month-old male mice was analyzed using μCT. (a) Representative reconstruction images are shown. (b-f) Histomorphometric parameters for cancellous bone were quantified, n=10. (g, h) Serum levels of PINP and CTX-1 were measured using ELISA, n=10. (i) ABH/OG staining was conducted. Scale bar: 200 μm (upper panel); 50 μm (lower panel). (j, k) The numbers and area percentage of adipocytes were quantified, n=6. (l) Representative images of osteocalcin IHC staining are shown. (m, n) The numbers and surface percentage of Ocn^+^ osteoblasts were quantified, n=6. (o) Representative images of TRAP staining are shown. (p, q) The numbers and surface percentage of osteoclasts were quantified, n=6. Scale in (l, o): 50 μm. Data are mean ± SD. Comparisons were conducted using Student’s t test, *p<0.05, **p<0.01, ***p<0.001; ns: no significance.

**
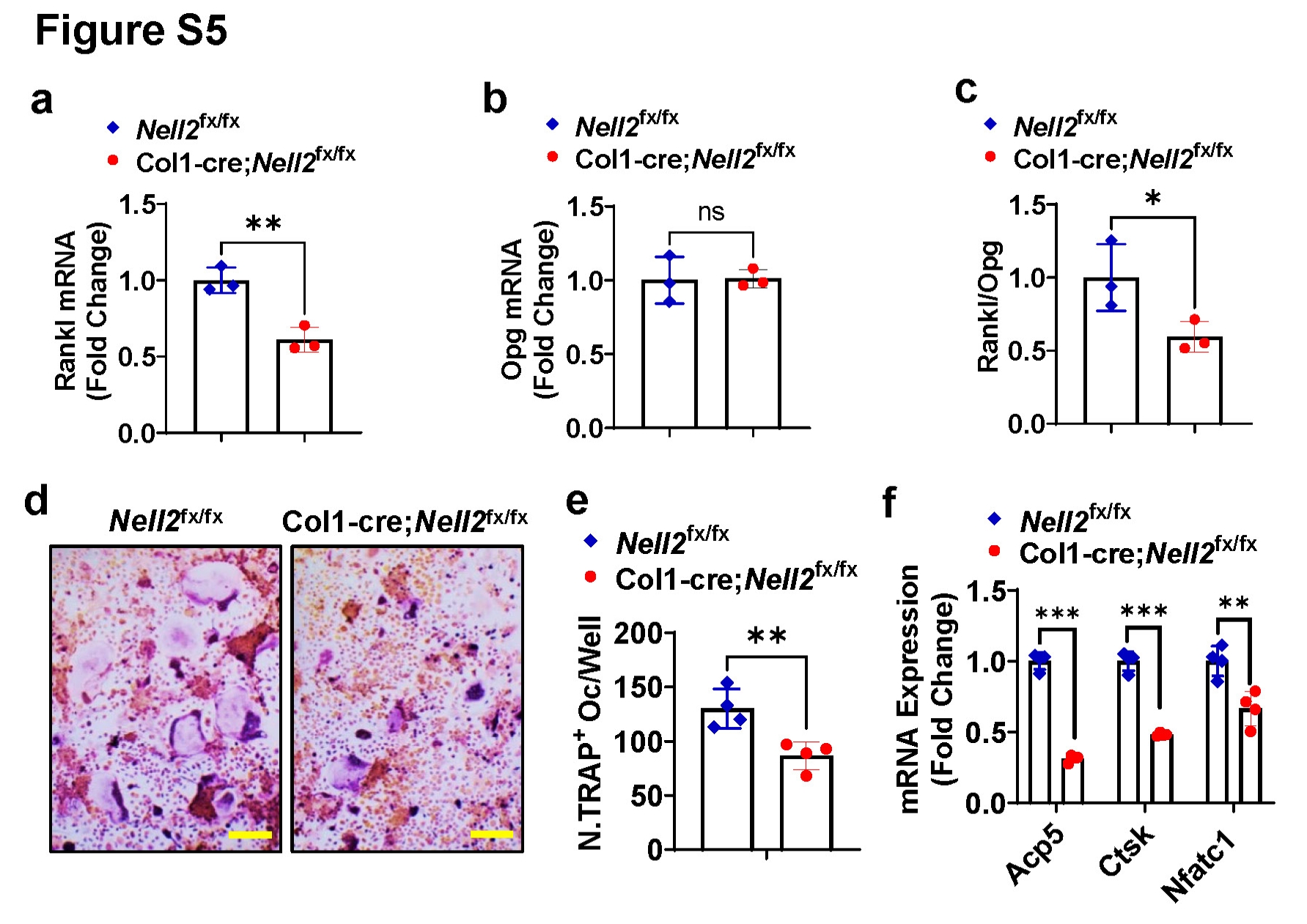
**

**Figure S5. Deletion of NELL2** **in preosteoblasts suppressed osteoclast differentiation.**

(a-c) Primary calvarial preosteoblasts from neonatal mice were cultured and the mRNA of Rankl and opg were detected, n=3. (d-f) Bone marrow cells were flushed from the femurs and tibiae of mice, cultured and induced to allow osteoclast differentiation. The cells were stained for TRAP (d) and the numbers of osteoclasts were counted (e), n=4. The marker genes for osteoclastogenesis were detected by qRT-PCR (f), n=4. Data are mean ± SD. Scale bar in (d): 100 μm. Comparisons were conducted using Student’s t test, *p<0.05, **p<0.01, ***p<0.001; ns: no significance.


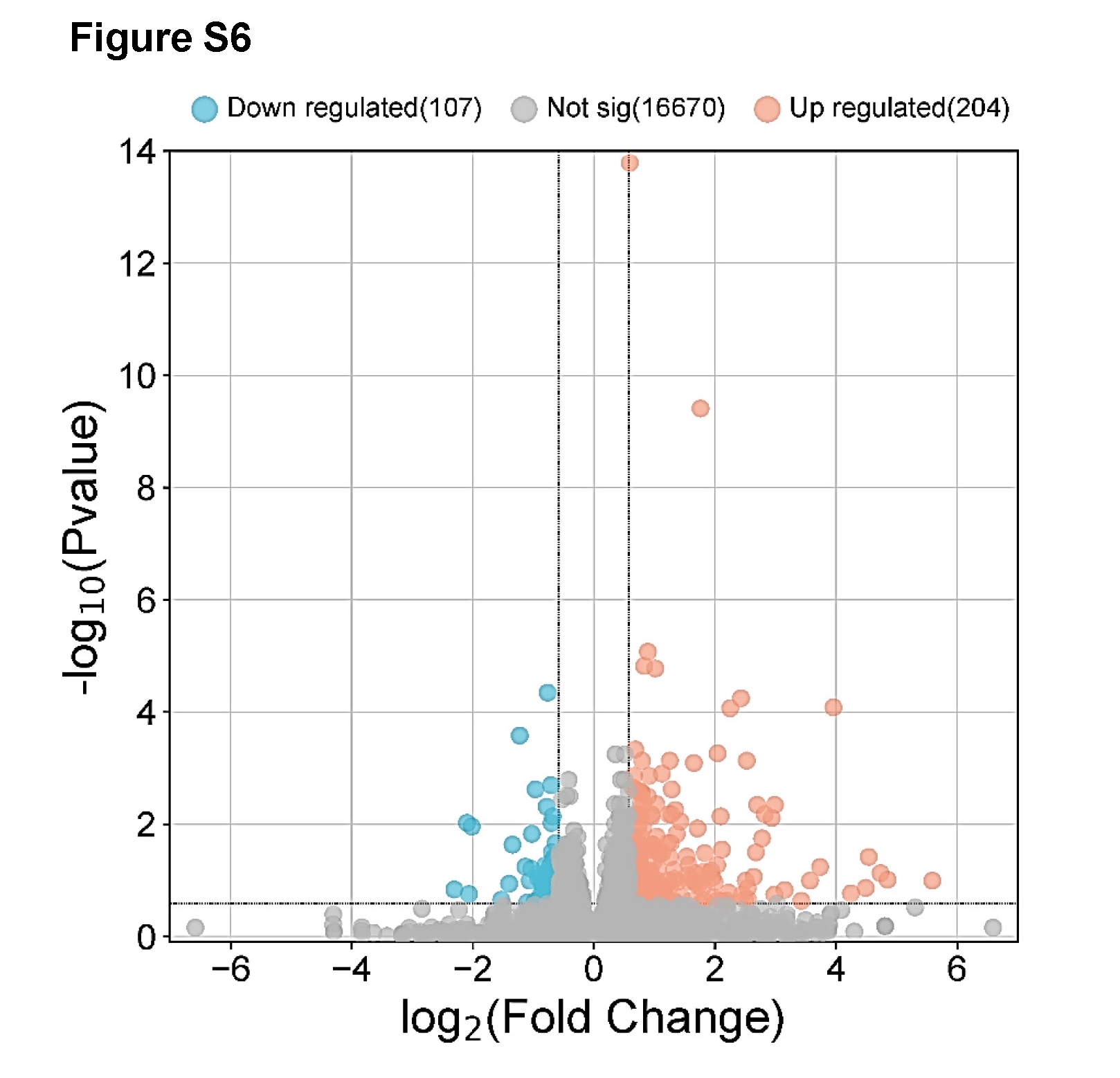


**Figure S6. Differentially expressed genes in preosteoblasts from Col1-cre;*Nell2*^fx/fx^ vs. *Nell2*^fx/fx^ mice are shown in the volcano plot.**

**
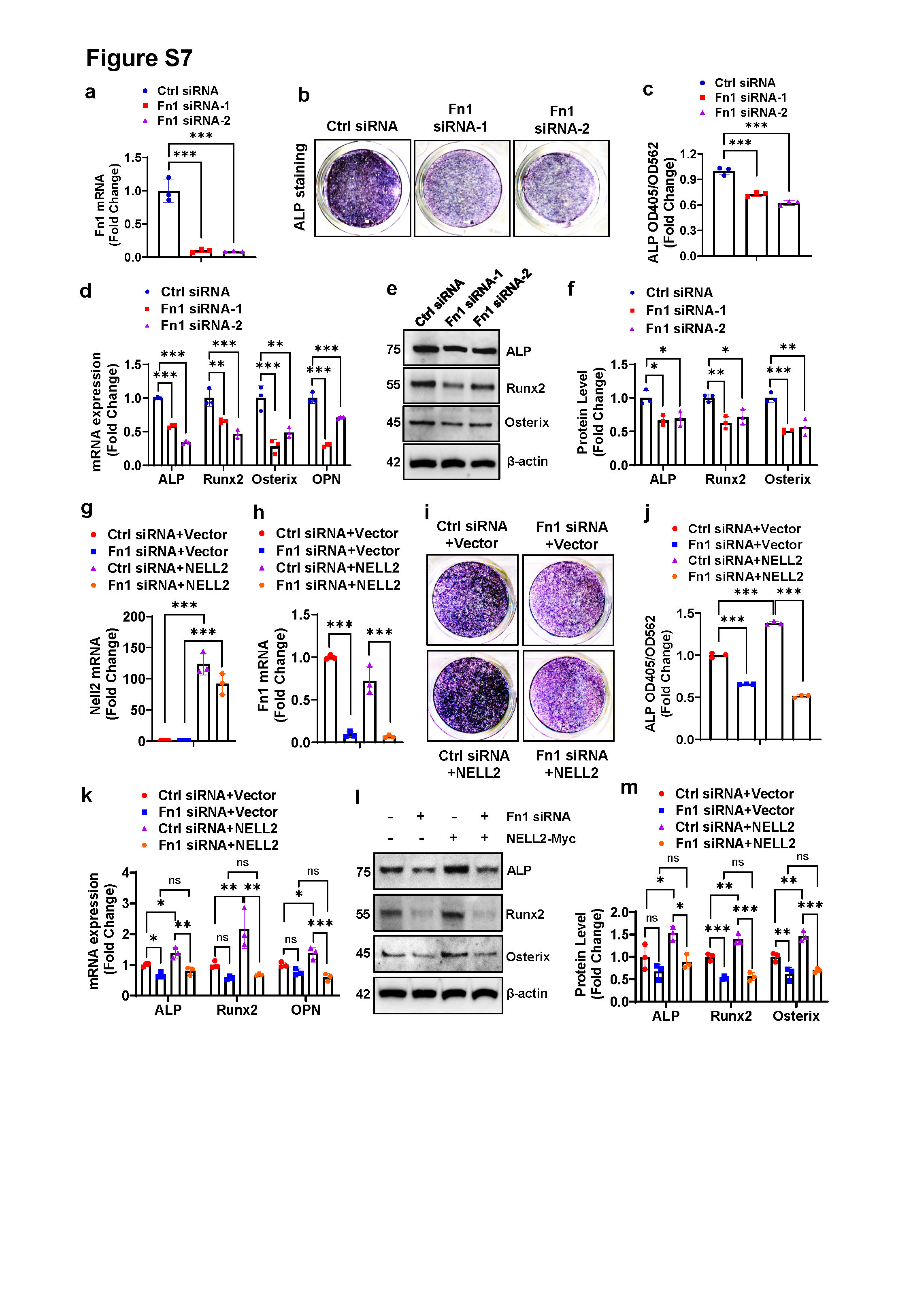
**

**Figure S7. Knockdown of Fn1 attenuated the stimulatory effect of NELL2 on osteoblast differentiation.**

ST2 cells were transfected with Fn1 siRNAs, and then induced to allow osteogenic differentiation. (a) qRT-PCR was performed to verify the knockdown of Fn1. (b) ALP staining was performed after 14 days of induction. (c) ALP activity was measured. (d-f) The mRNA and protein levels of osteogenic factors were detected using qRT-PCR and Western blotting. ST2 cells were co-transfected with NELL2-Myc construct (or vector) and Fn1 siRNA(siRNA-2) (or control siRNA), and then induced to allow osteogenic differentiation. (g, h) qRT-PCR was performed to verify NELL2 overexpression (g) and Fn1 knockdown (h). (i) ALP staining was performed. (j) ALP activity was measured. (k-m) The mRNA and protein levels of osteogenic factors were detected. Data are mean ± SD, n=3. Comparisons were conducted using one-way ANOVA followed by Dunnett’s test (a, c, d, f), or two-way ANOVA followed by Tukey’s test (g, h, j, k, m), *p<0.05, **p<0.01, ***p<0.001; ns: no significance.

**
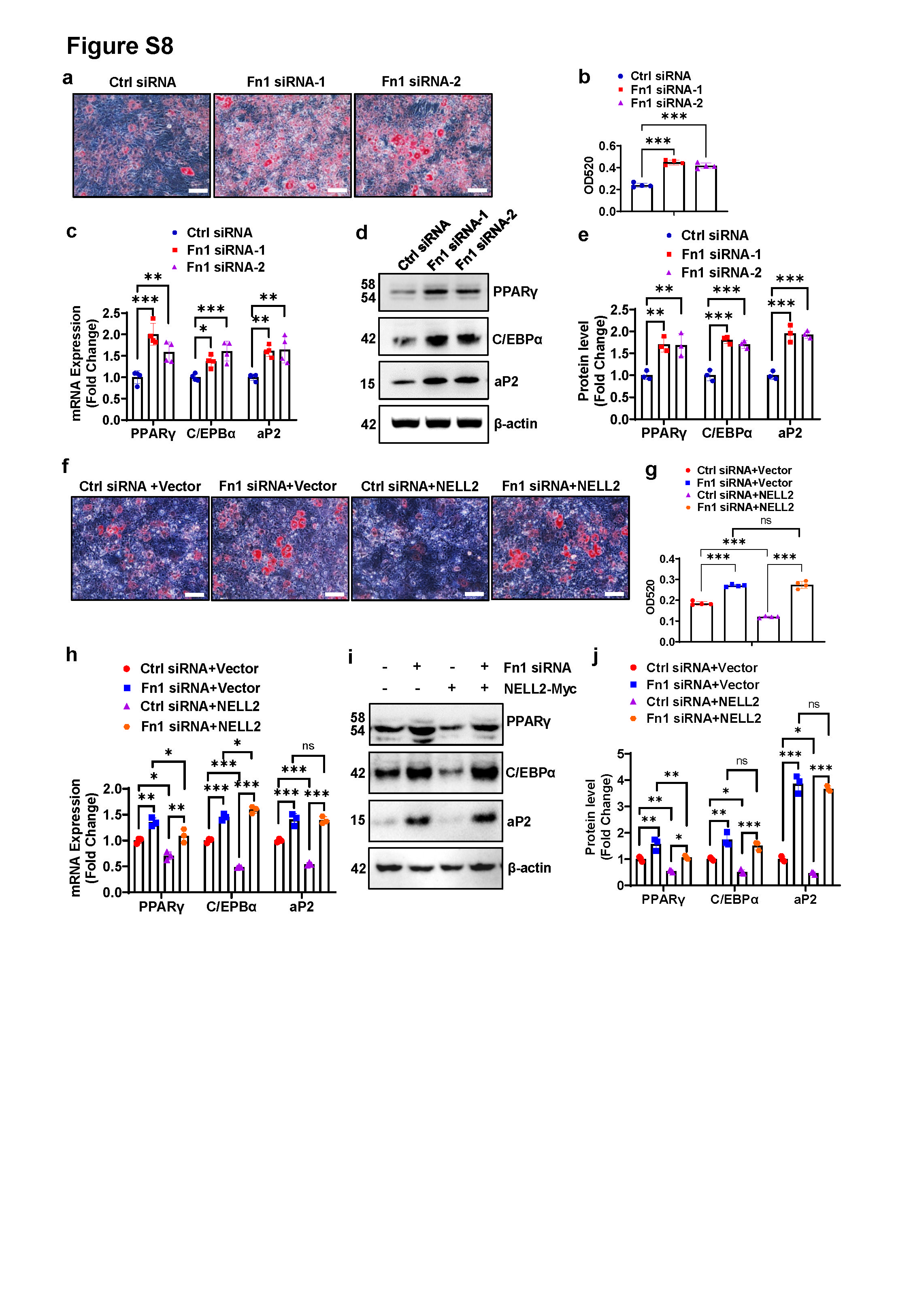
**

**Figure S8. Knockdown of Fn1 mitigated the inhibitory effect of NELL2 on adipocyte differentiation.**

ST2 cells were transfected with Fn1 siRNAs, and then induced to allow adipogenic differentiation. (a) Differentiated adipocytes were stained with oil red O after 5-6 days of induction. (b) Oil red O extracted with isopropanol was measured at OD520, n=4. (c-e) The mRNA (n=4) and protein (n=3) levels of adipogenic factors were examined. ST2 cells were co-transfected with NELL2-Myc construct (or vector) and Fn1 siRNA (or control siRNA), and then induced to allow adipogenic differentiation. (f) Differentiated adipocytes were stained with oil red O. (g) Oil red O extracted with isopropanol was measured at OD520, n=4. (h-j) The mRNA and protein levels of adipogenic factors were examined, n=3. Scale in (a, f): 100 μm. Data are mean ± SD. Comparisons were conducted using one-way ANOVA followed by Dunnett’s test (b, c, e), or two-way ANOVA followed by Tukey’s test (g, h, j), *p<0.05, **p<0.01, ***p<0.001; ns: no significance.

**
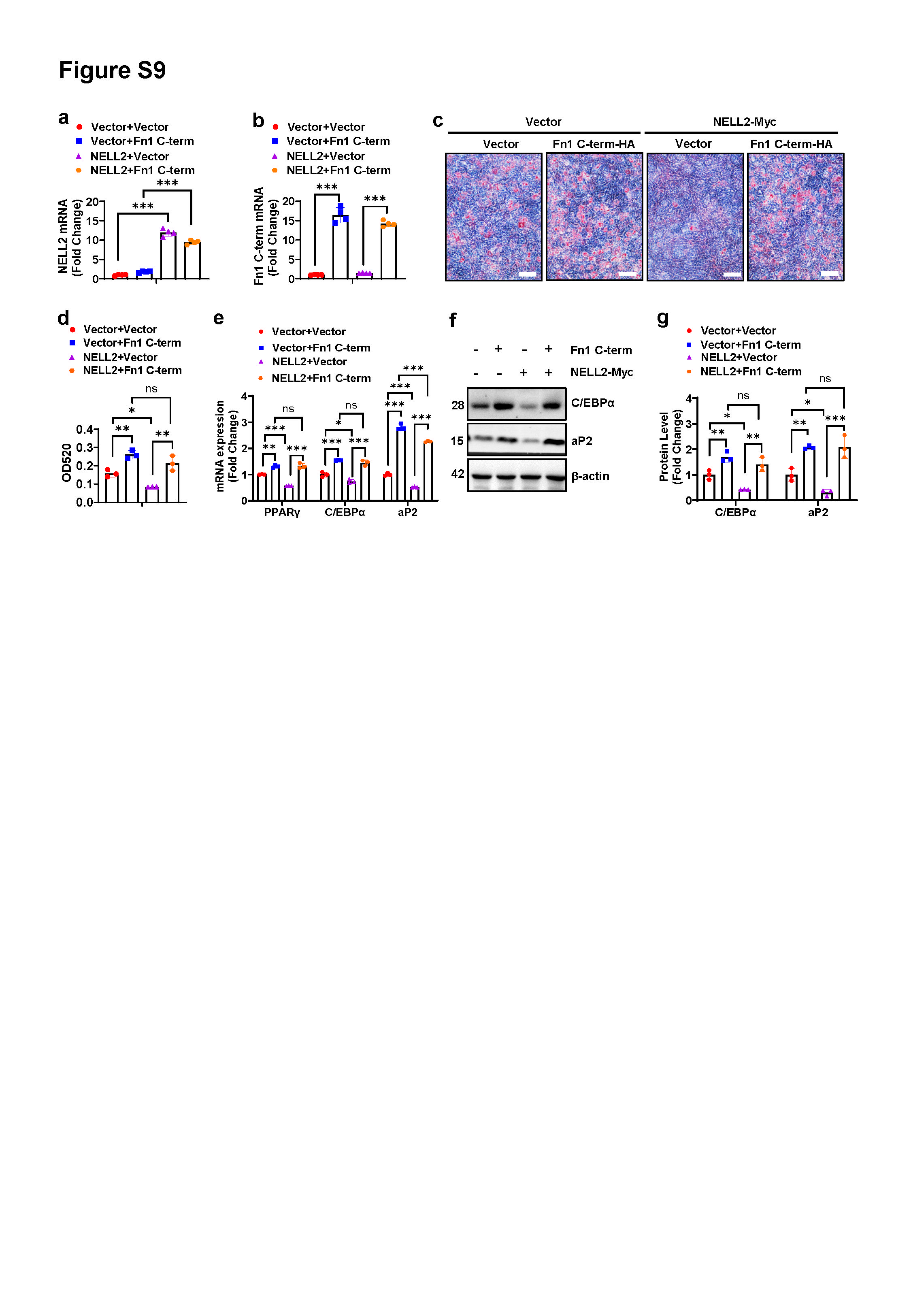
**

**Figure S9. Overexpression of Fn1 C-terminal mitigated the inhibitory effect of NELL2 on adipocyte differentiation.**

ST2 cells were co-transfected with NELL2-Myc (or vector) and Fn1 C-terminal-HA constructs (or vector), and then induced to allow adipogenic differentiation. (a, b) qRT-PCR was performed to verify NELL2 overexpression (a, n=4) and Fn1 C-terminal overexpression (b, n=4). (c) Differentiated adipocytes were stained with oil red O after 5-6 days of induction. (d) Oil red O extracted with isopropanol was measured at OD520, n=3. (e-g) The mRNA and protein levels of adipogenic factors were examined (n=3). Scale bar in (c): 100 μm. Data are mean ± SD. Comparisons were conducted using two-way ANOVA followed by Tukey’s test, *p<0.05, **p<0.01, ***p<0.001; ns: no significance.

**
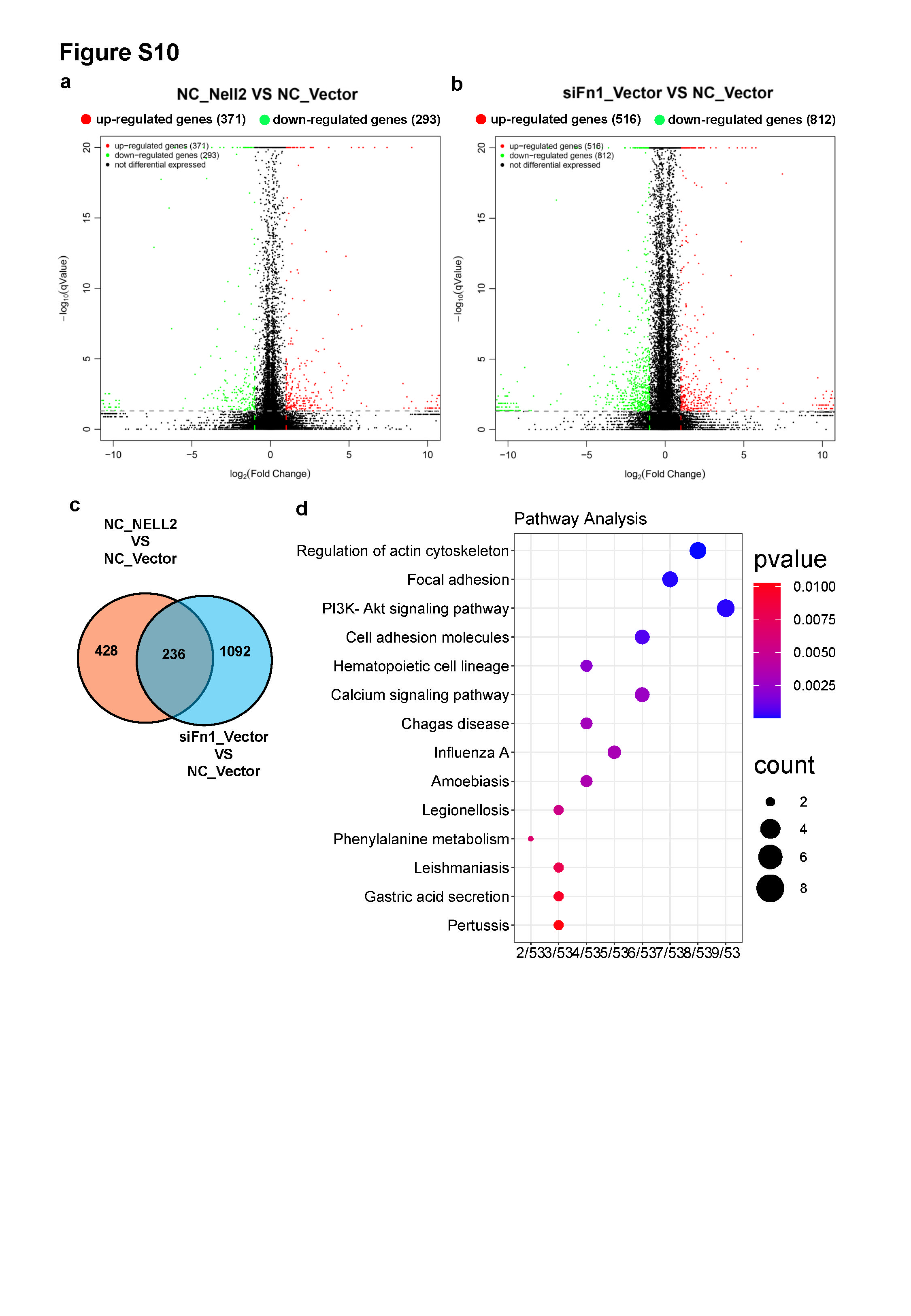
**

**Figure S10. Genes co-regulated by NELL2 and Fn1 were identified through RNA-seq analysis.**

(a, b) The differentially expressed genes in ST2 cells transfected with NELL2-Myc construct (or vector) (a) or Fn1 siRNA (or control siRNA) (b) were identified by RNA-Seq and the volcano maps are presented. (c) A Venn diagram is presented to illustrate the number of overlapping altered genes by NELL2 and Fn1. (d) KEGG analysis was performed to identify enriched pathways associated with the overlapping altered genes.

**
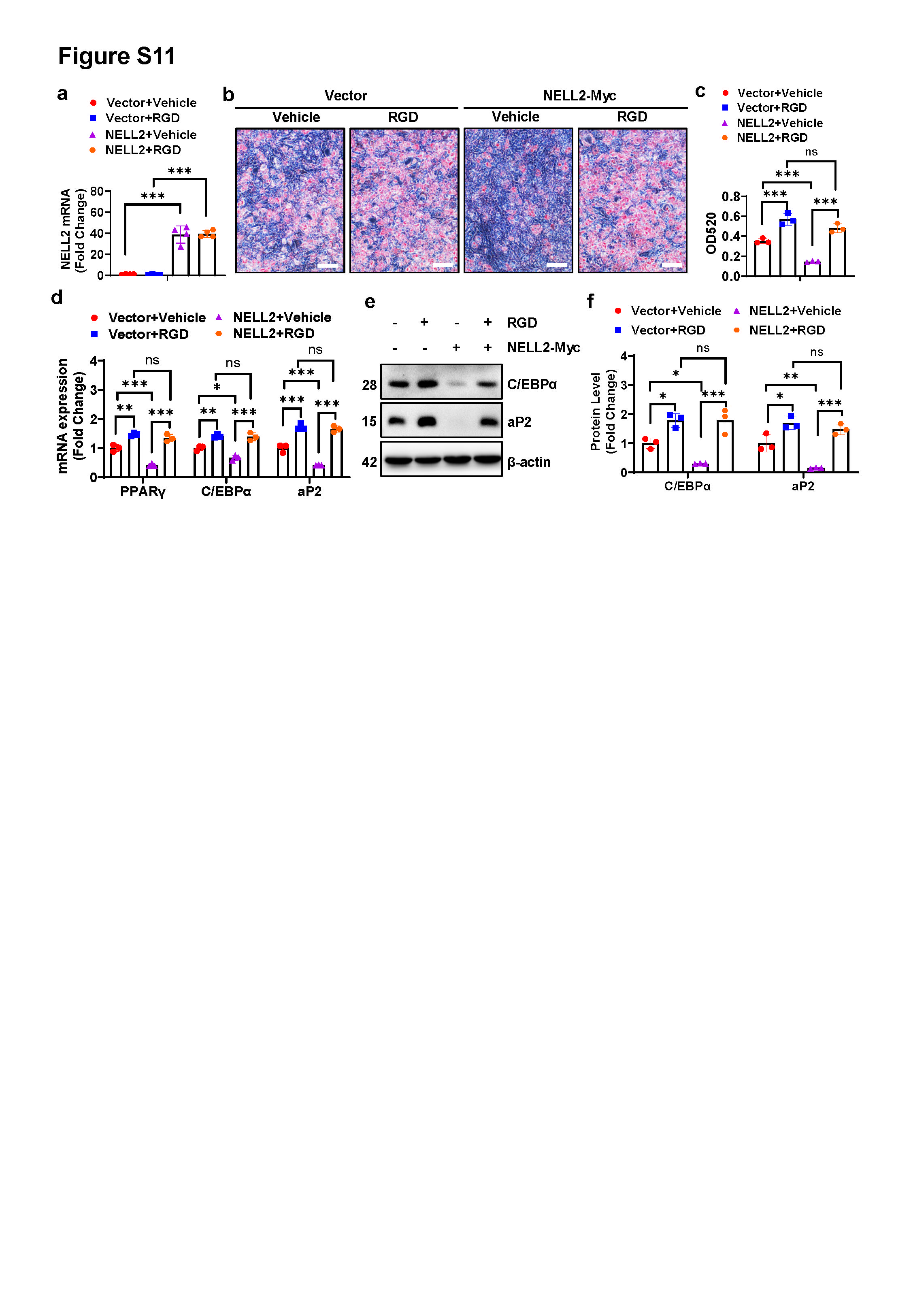
**

**Figure S11. RGD peptide mitigated the inhibitory effect of NELL2 on adipocyte differentiation.**

ST2 cells were transfected with NELL2-Myc (or vector), and then induced to allow adipogenic differentiation in the presence of vehicle or 10 μM RGD peptide (GRGDSPK). (a) qRT-PCR was performed to verify NELL2 overexpression (n=4). (b) Differentiated adipocytes were stained with oil red O after 5-6 days of induction. (c) Oil red O extracted with isopropanol was measured at OD520, n=3. (d-f) The mRNA and protein levels of adipogenic factors were examined (n=3). Scale in (b): 100 μm. Data are mean ± SD. Comparisons were conducted using two-way ANOVA followed by Tukey’s test, *p<0.05, **p<0.01, ***p<0.001; ns: no significance.

**
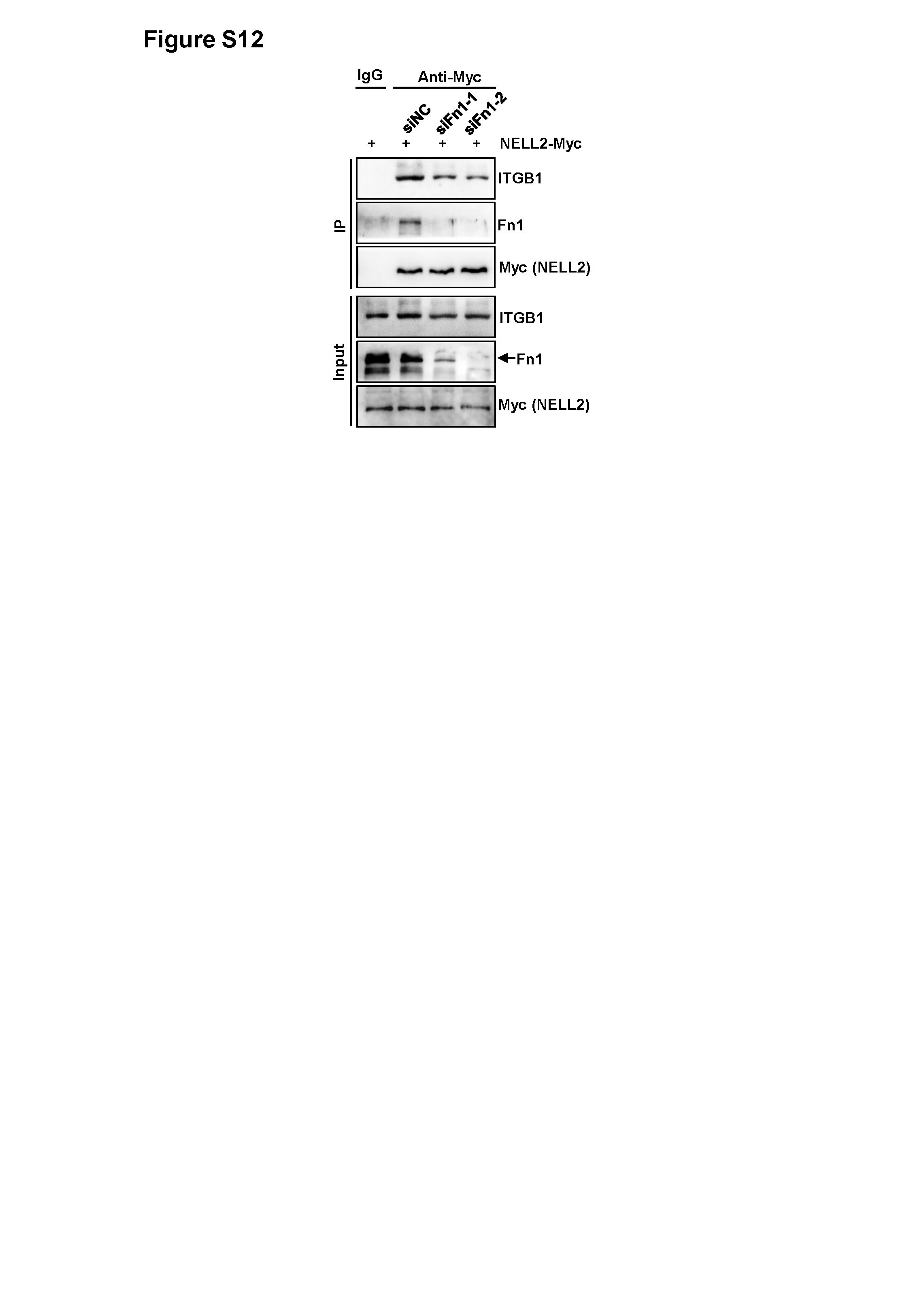
**

**Figure S12. Knockdown of Fn1 reduced NELL2-ITGB1 interaction.**

ST2 cells co-transfected with NELL2-Myc plus Fn1 siRNAs or control siRNA. The cell lysates were immunoprecipitated with anti-Myc and Western blotting was performed to detect the presence of NELL2-Myc, Fn1 and ITGB in the immunoprecipitates. The proteins were also detected in the input.

**
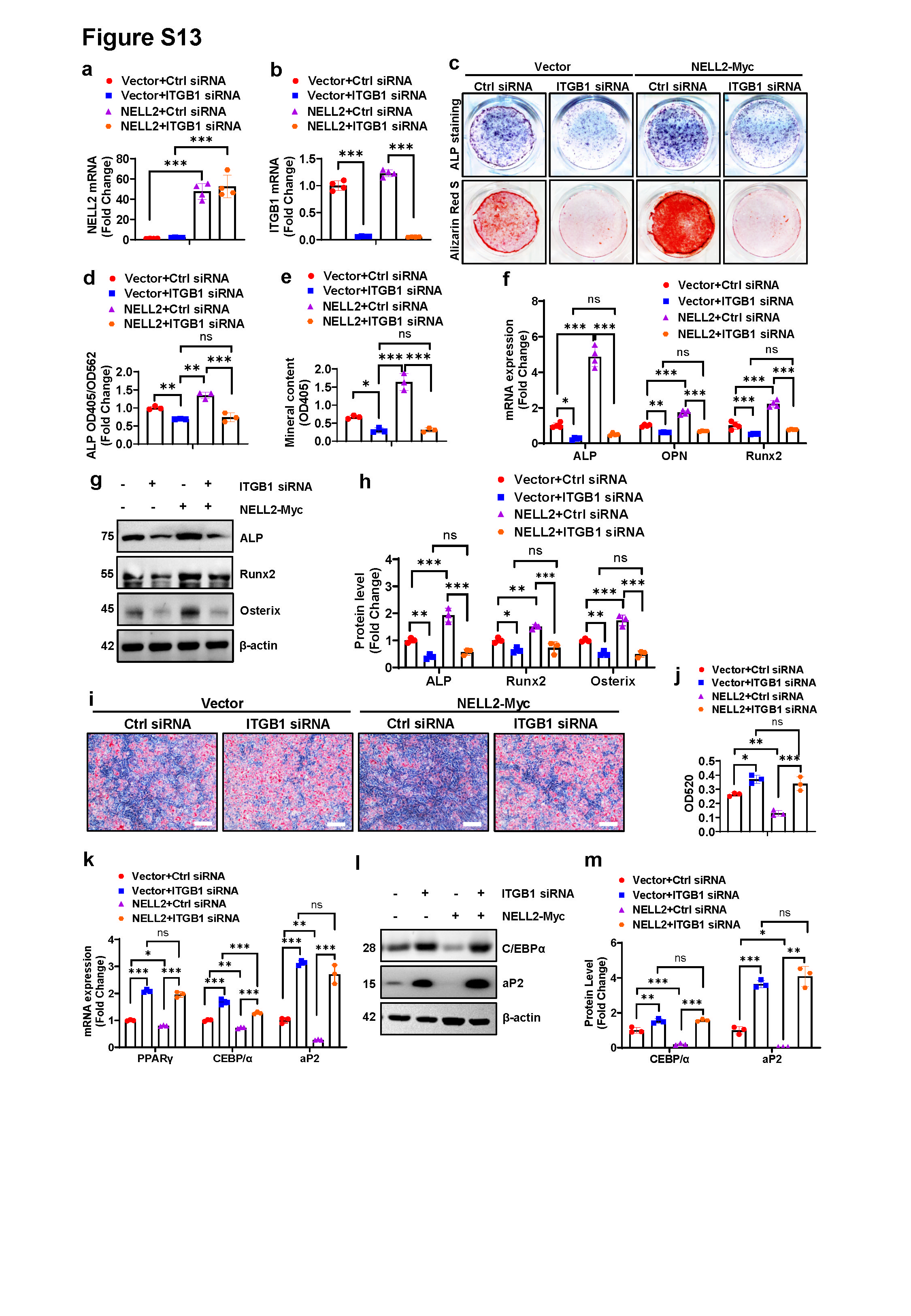
**

**Figure S13.** **Knockdown of ITGB1 mitigated NELL2-induced effects on osteoblast and adipocyte differentiation.**

ST2 cells were co-transfected with NELL2-Myc construct (or vector) and ITGB1 siRNA (or control siRNA), and then induced to allow osteogenic differentiation (a-h) or adipocyte differentiation (i-m). (a, b) qRT-PCR was performed to verify NELL2 overexpression (a) and ITGB1 knockdown (b), n=4. (c) ALP staining (upper panel) and alizarin red S staining (lower panel) were performed after 14 and 21 days of induction, respectively. (d) ALP activity was measured. (e) The intensity of alizarin red S staining was quantified. (f-h) The mRNA (f) and protein (g, h) levels of osteogenic factors were detected after 3 days of induction. n=3. (i) Differentiated adipocytes were stained with oil red O after 5-6 days of induction. (j) Oil red O extracted with isopropanol was measured at OD520. (k-m) The mRNA and protein levels of adipogenic factors were examined. n=3. Scale: 100 μm. Data are mean ± SD. Comparisons were conducted using two-way ANOVA followed by Tukey’s test, *p<0.05, **p<0.01, ***p<0.001; ns: no significance.

**
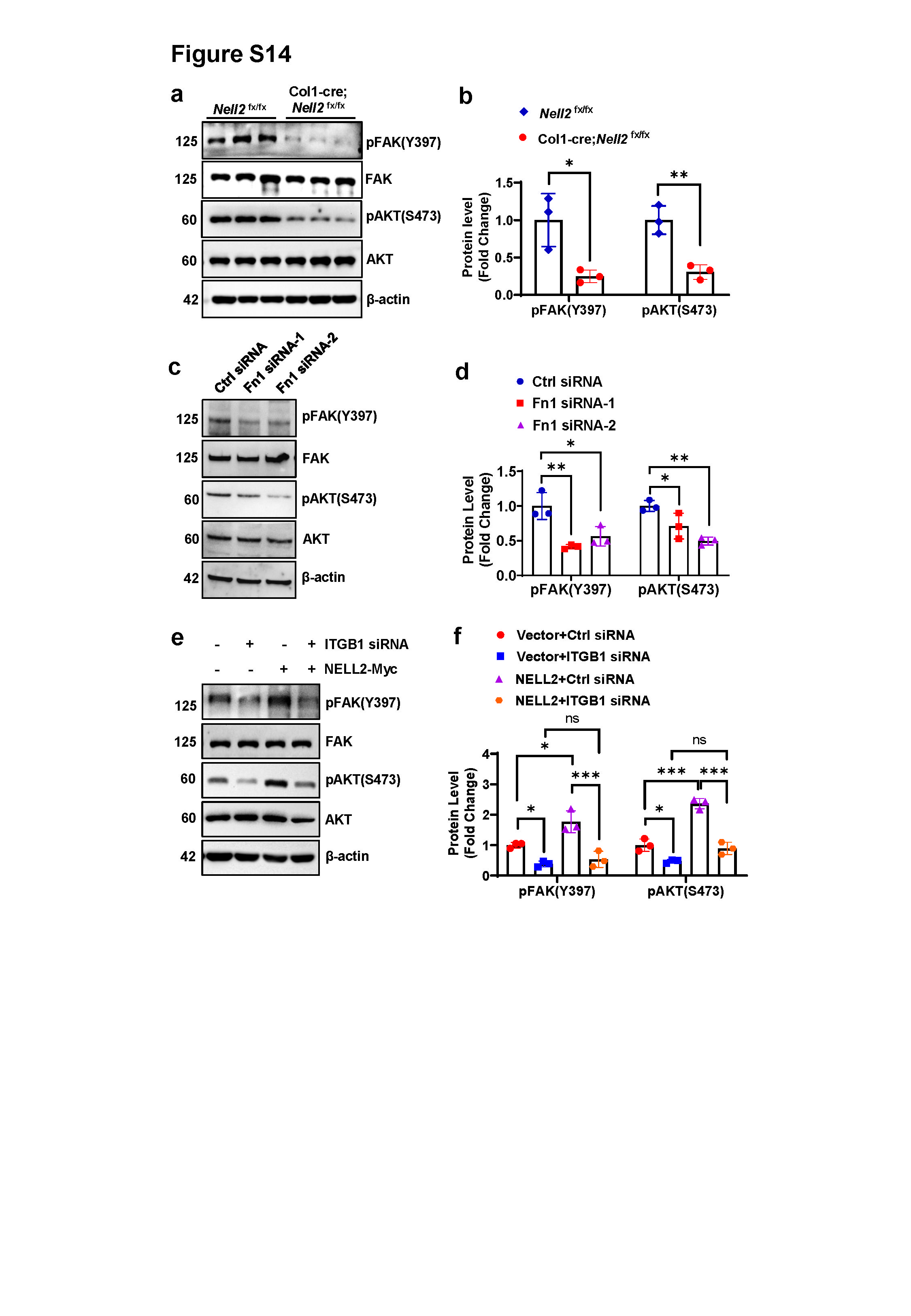
**

**Figure S14. NELL2 activated FAK/AKT signaling pathway through Fibronectin 1/integrin.**

(a, b) Western blotting was conducted to analyze pFAK(Y397), FAK, pAKT(S473) and AKT levels in preosteoblasts from Col1-cre;*Nell2*^fx/fx^ mice compared to *Nell2*^fx/fx^ mice. (c, d) Western blotting was conducted to analyze pFAK(Y397), FAK, pAKT(S473) and AKT levels in ST2 cells transfected with with Fn1 siRNAs (or control siRNA). (e, f) Western blotting was conducted to analyze pFAK(Y397), FAK, pAKT (S473) and AKT levels in ST2 cells co-transfected with NELL2-Myc construct (or vector) and ITGB1 siRNA (or control siRNA). Data are mean ± SD, n=3. Comparisons were conducted using Student’s t test (b), one-way ANOVA followed by Dunnett’s test (d), or two-way ANOVA followed by Tukey’s test (f). *p<0.05, **p<0.01, ***p<0.001; ns: no significance.

**
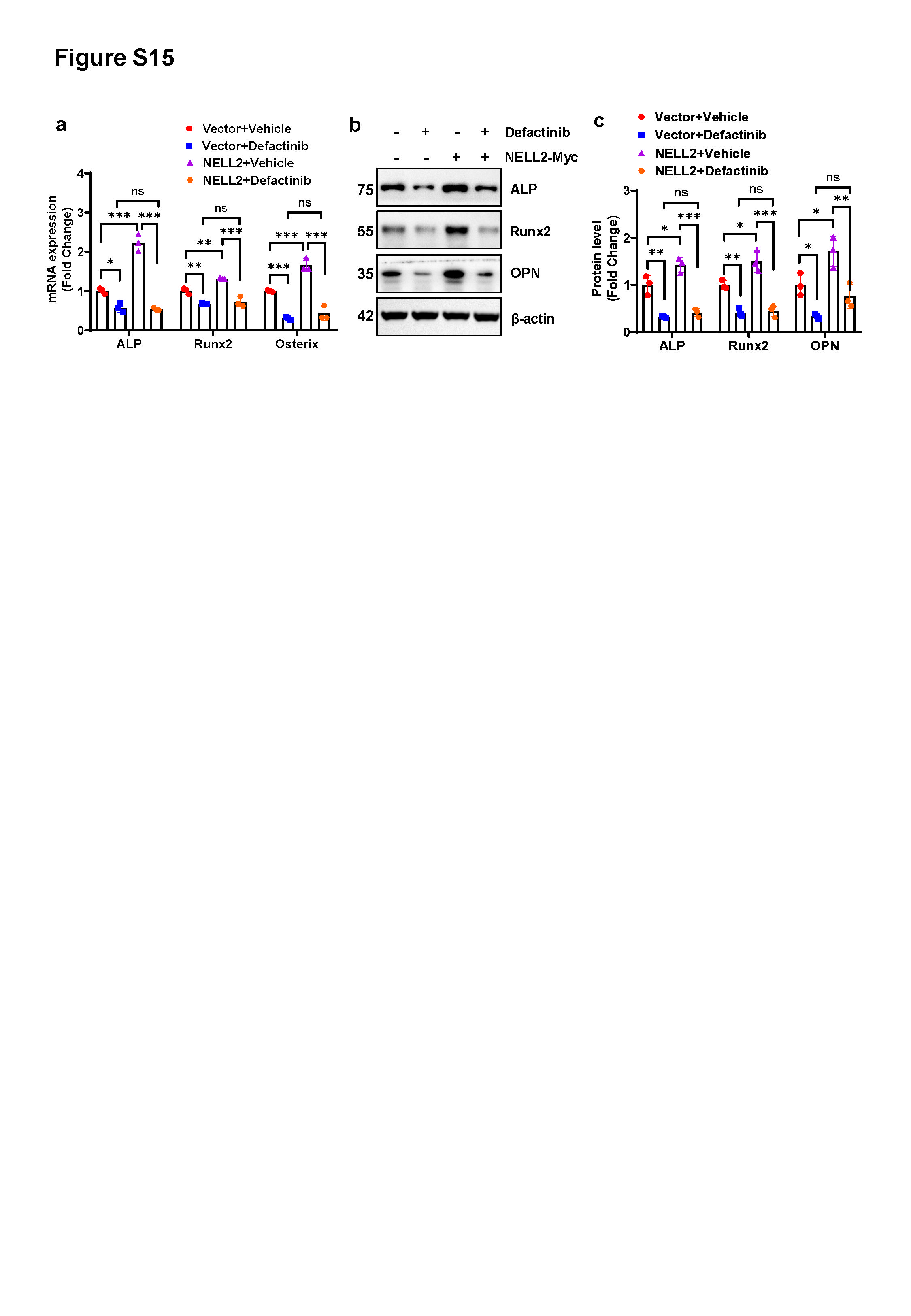
**

**Figure S15. FAK inhibitor mitigated the** **pro-osteogenic effect of NELL2 in ST2 cells.**

ST2 cells were transfected with NELL2-Myc (or vector), and then induced to allow osteogenic differentiation in the presence of Vehicle or 1 μM Defactinib. (a-c) The mRNA (a) and protein (b, c) levels of osteogenic factors were detected after 3 days of induction. Data are mean ± SD, n=3. Comparisons were conducted using two-way ANOVA followed by Tukey’s test. *p<0.05, **p<0.01, ***p<0.001; ns: no significance.

**
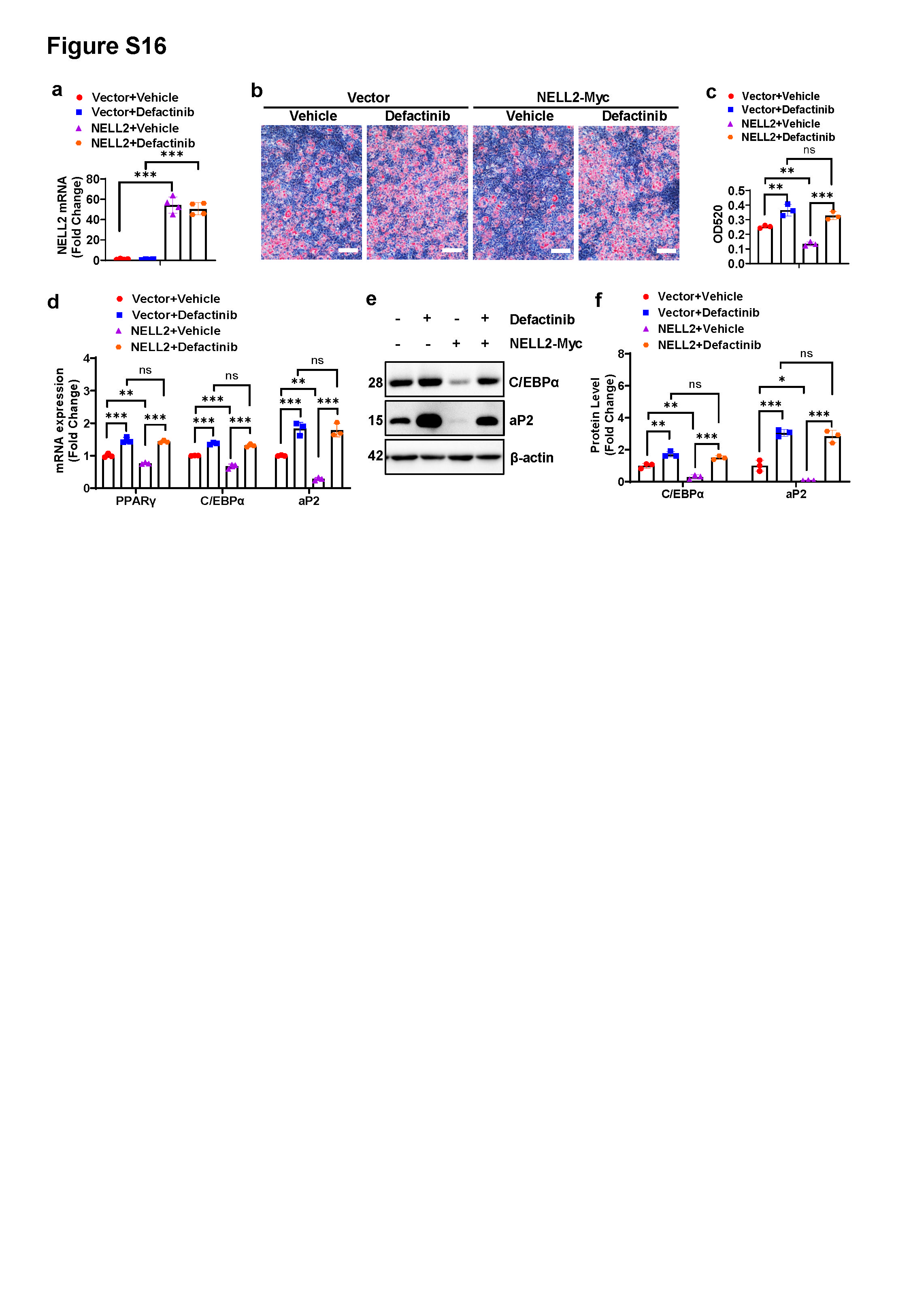
**

**Figure S16. FAK inhibitor mitigated the anti-adipogenic effect of NELL2 in ST2 cells.**

ST2 cells were transfected with NELL2-Myc (or vector), and then induced to allow adipogenic differentiation in the presence of Vehicle or 1 μM Defactinib. (a) qRT-PCR was performed to verify NELL2 overexpression (n=4). (b) Differentiated adipocytes were stained with oil red O after 5-6 days of induction. (c) Oil red O extracted with isopropanol was measured at OD520, n=3. (d-f) The mRNA and protein levels of adipogenic factors were examined (n=3). Scale in (b):100 μm. Data are mean ± SD. Comparisons were conducted using two-way ANOVA followed by Tukey’s test, *p<0.05, **p<0.01, ***p<0.001; ns: no significance.

**
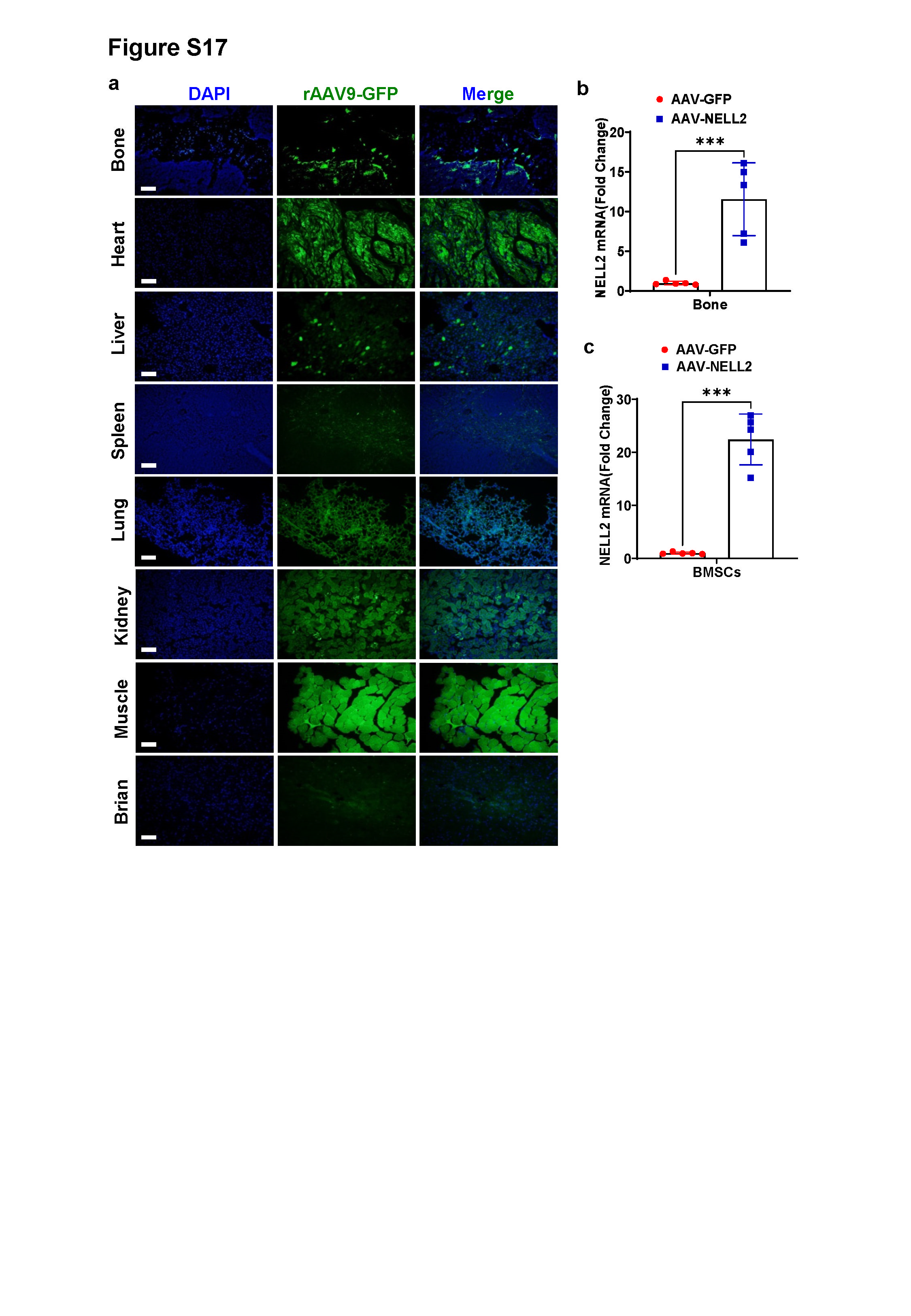
**

**Figure S17. NELL2-AAV was effective in transducing bone.**

(a). One month after AAV delivery, mice were sacrificed and tissue distribution of NELL2-AAV was examined by observing cryosections under fluorescence microscope. Representative fluorescent images of various tissues are shown. Scale bar: 100 μm. (b, c) qRT-PCR was conducted to confirm overexpression of NELL2 in bone (b) and BMSCs (c). Data are mean ± SD, n=5. Comparisons were conducted using Student’s t test, ***p<0.001.

**
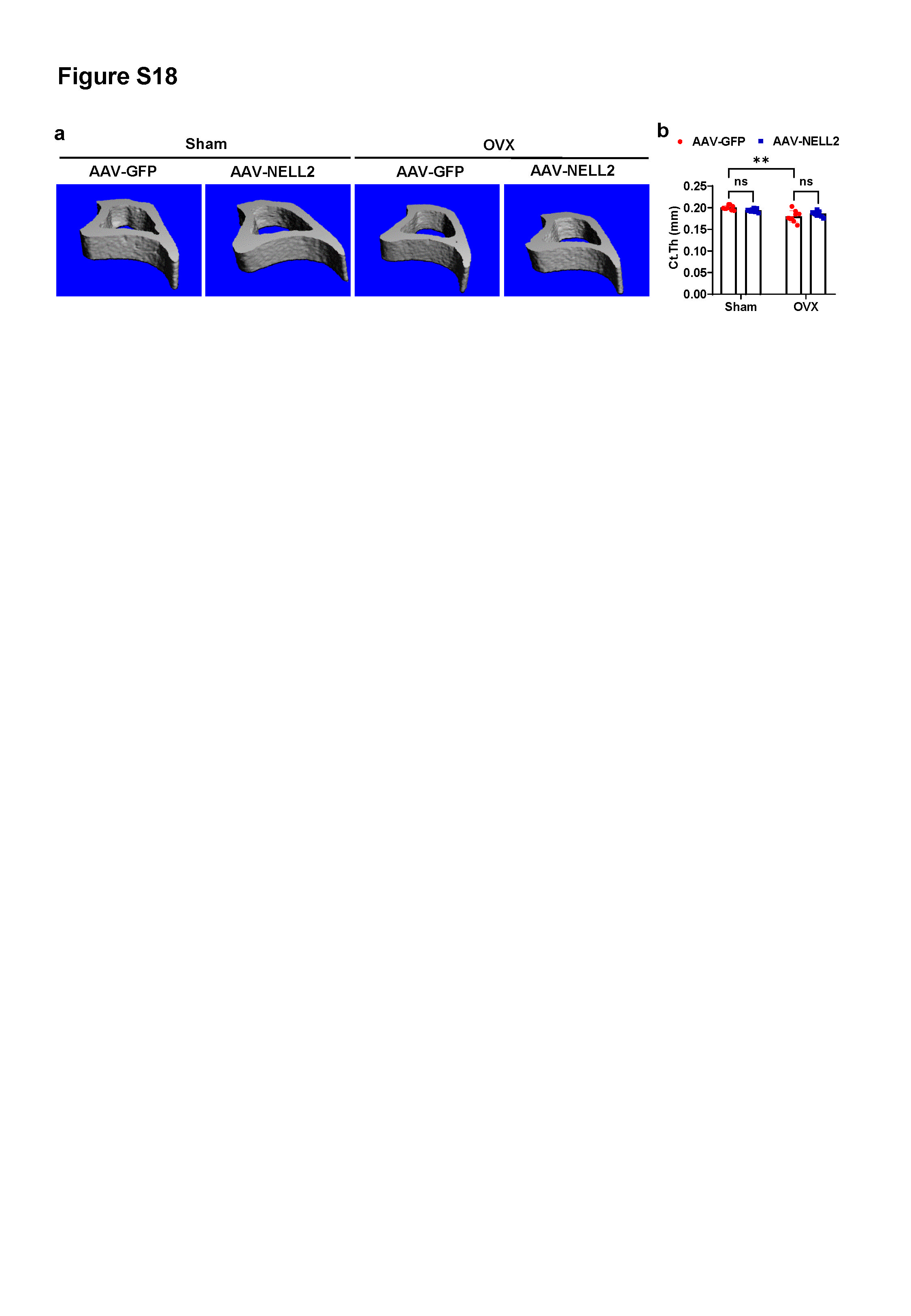
**

**Figure S18. Administration NELL2-AAV had no effect on cortical bone in OVX mice.**

(a) Representative images of tibial cortical bone are shown. (b) Cortical thickness was measured on the tibiae of the mice, n=8-9. Data are mean ± SD. Comparisons were conducted using two-way ANOVA followed by Tukey’s test, **p<0.01; ns: no significance.


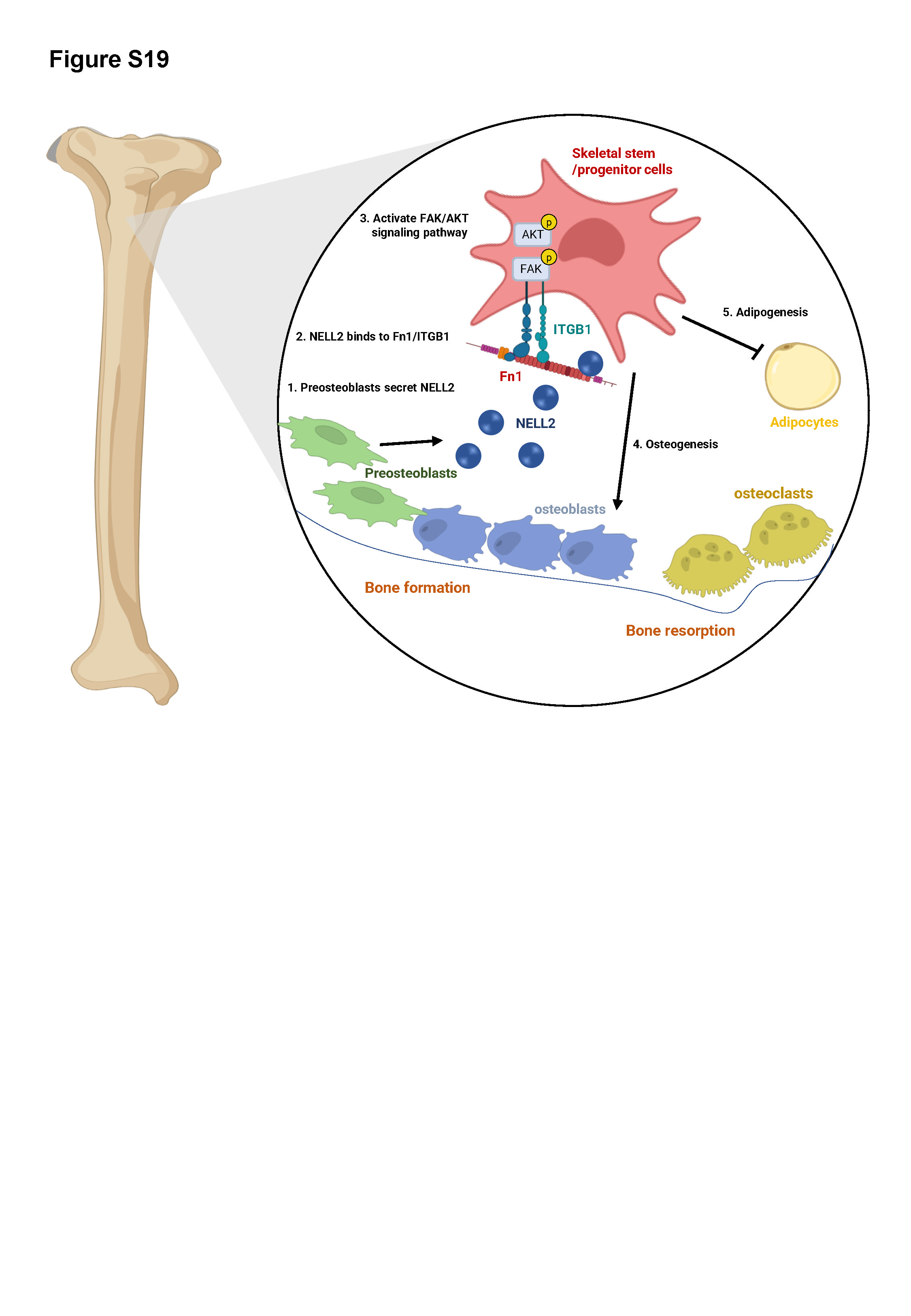


**Figure S19. Schematic model illustrating the role of NELL2 in osteoblast differentiation and bone homeostasis.**

(1) Preosteoblasts secret NELL2. (2) NELL2 interacts with Fn1/ITGB1 to activate the integrin-mediated FAK/AKT signaling pathway (3), thereby promoting osteoblast differentiation and bone formation (4), while simultaneously inhibiting marrow adipocyte formation (5).

Table S1. Demographic and clinical characteristics of the postmenopausal women in the study.

|  | Normal bone mass(n=5) | Osteopenia  (n=13) | Osteoporosis  (n=10) | F Value | p Value |
| --- | --- | --- | --- | --- | --- |
| Age, year | 59.94±3.26 | 63.87±5.08 | 65.1±4.39 | 2.623 | 0.093 |
| BMI, kg/m^2^ | 29.38±3.49 | 26.64±3.51 | 24.54±2.52 | 3.916 | 0.033 ^b^ |
| Menopause age, year | 49.80±3.19 | 50.00±2.48 | 49.50+4.77 | 0.055 | 0.946 |
| Femur neck BMD, g/cm^2^ | 1.00±0.05 | 0.82±0.07 | 0.69±0.06 | 38.52 | <0.001 ^a,b,c^ |
| Total hip BMD, g/cm^2^ | 1.08±0.08 | 0.87±0.08 | 0.77±0.06 | 29.11 | <0.001 ^a,b,c^ |
| Lumbar spine BMD(L1-L4), g/cm^2^ | 1.31±0.15 | 1.08±0.17 | 0.91±0.12 | 11.59 | <0.001 ^a,b,c^ |
| Femur neck T score | -0.22±0.35 | -1.55±0.55 | -2.51±0.42 | 38.57 | <0.001 ^a,b,c^ |
| Total hip T score | 0.58±0.65 | -1.06±0.63 | -1.07±1.05 | 28.37 | <0.001 ^a,b,c^ |
| Lumbar spine T score(L1-L4) | 1.10±1.29 | -0.67±1.35 | -2.18±1.01 | 12.91 | <0.001 ^a,b,c^ |

^a^ The difference between normal bone mass and osteopenia group was statistically significant

^b^ The difference between normal bone mass and osteoporosis group was statistically significant

^c^ The difference between osteopenia and osteoporosis group was statistically significant

Table S2 MS list.

| Accession | GeneName | Coverage [%] | # Peptides | # PSMs | # Unique Peptides | # AAs | MW [kDa] | calc. pI | Score |
| --- | --- | --- | --- | --- | --- | --- | --- | --- | --- |
| Q61220 | Nell2 | 28 | 22 | 133 | 22 | 819 | 91.4 | 5.82 | 355.43 |
| P11276 | Fn1 | 30 | 48 | 69 | 48 | 2477 | 272.4 | 5.59 | 230.09 |
| Q03265 | Atp5f1a | 25 | 10 | 12 | 10 | 553 | 59.7 | 9.19 | 36.57 |
| P26443 | Glud1 | 23 | 10 | 12 | 10 | 558 | 61.3 | 8 | 32.16 |
| Q9Z110 | Aldh18a1 | 17 | 11 | 12 | 11 | 795 | 87.2 | 7.55 | 32.1 |
| Q9DCN2 | Cyb5r3 | 20 | 5 | 9 | 5 | 301 | 34.1 | 8.38 | 31.2 |
| Q60932 | Vdac1 | 47 | 10 | 11 | 9 | 296 | 32.3 | 8.43 | 30.33 |
| Q02257 | Jup | 12 | 7 | 8 | 7 | 745 | 81.7 | 6.14 | 22.48 |
| Q99PL5 | Rrbp1 | 6 | 7 | 7 | 7 | 1605 | 172.8 | 9.33 | 21.13 |
| O35129 | Phb2 | 32 | 8 | 8 | 8 | 299 | 33.3 | 9.83 | 20.17 |
| Q9DBV4 | Mxra8 | 15 | 6 | 6 | 6 | 442 | 49.7 | 7.15 | 17.38 |
| P38647 | Hspa9 | 11 | 6 | 7 | 6 | 679 | 73.4 | 6.07 | 16.84 |
| Q8BMS1 | Hadha | 11 | 6 | 6 | 6 | 763 | 82.6 | 9.14 | 16.05 |
| Q8K0E8 | Fgb | 13 | 5 | 6 | 5 | 481 | 54.7 | 7.08 | 15.74 |
| P30999 | Ctnnd1 | 8 | 6 | 6 | 6 | 938 | 104.9 | 6.87 | 15.58 |
| Q60931 | Vdac3 | 16 | 4 | 5 | 3 | 283 | 30.7 | 8.79 | 15.56 |
| P08752 | Gnai2 | 15 | 4 | 5 | 4 | 355 | 40.5 | 5.45 | 15.55 |
| Q9CZN7 | Shmt2 | 13 | 5 | 5 | 5 | 504 | 55.7 | 8.47 | 14.97 |
| Q91V41 | Rab14 | 30 | 4 | 5 | 3 | 215 | 23.9 | 6.21 | 14.74 |
| Q9D379 | Ephx1 | 15 | 5 | 5 | 5 | 455 | 52.5 | 8.35 | 14.59 |
| P07724 | Alb | 10 | 5 | 6 | 5 | 608 | 68.6 | 6.07 | 14.58 |
| P11589 | Mup2 | 38 | 5 | 5 | 1 | 180 | 20.7 | 5.11 | 14.42 |
| P36369 | Klk1b26 | 16 | 3 | 4 | 3 | 261 | 28.4 | 6.86 | 14.16 |
| P47738 | Aldh2 | 9 | 4 | 5 | 4 | 519 | 56.5 | 7.62 | 13.45 |
| Q9ESB3 | Hrg | 9 | 5 | 5 | 5 | 525 | 59.1 | 7.66 | 13.41 |
| Q8VCM7 | Fgg | 16 | 5 | 5 | 5 | 436 | 49.4 | 5.86 | 13.2 |
| Q8BH59 | Slc25a12 | 10 | 4 | 4 | 4 | 677 | 74.5 | 8.25 | 13.19 |
| Q91YQ5 | Rpn1 | 10 | 5 | 5 | 5 | 608 | 68.5 | 6.46 | 13.04 |
| Q31125 | Slc39a7 | 11 | 3 | 4 | 3 | 476 | 50.6 | 6.87 | 13.01 |
| P15949 | Klk1b9 | 13 | 2 | 4 | 2 | 261 | 28.9 | 7.64 | 12.7 |
| Q8BMK4 | Ckap4 | 10 | 5 | 5 | 5 | 575 | 63.7 | 5.64 | 12.51 |
| Q9DBG6 | Rpn2 | 11 | 4 | 4 | 4 | 631 | 69 | 5.81 | 12.46 |
| O54734 | Ddost | 11 | 4 | 4 | 4 | 441 | 49 | 5.83 | 12.36 |
| P63001 | Rac1 | 24 | 5 | 5 | 5 | 192 | 21.4 | 8.5 | 11.99 |
| P62874 | Gnb1 | 18 | 5 | 5 | 5 | 340 | 37.4 | 6 | 11.49 |
| Q6P5F6 | Slc39a10 | 6 | 5 | 5 | 5 | 833 | 94.3 | 6.71 | 11.48 |
| P55258 | Rab8a | 21 | 4 | 5 | 2 | 207 | 23.7 | 9.07 | 11.25 |
| E9PV24 | Fga | 5 | 3 | 4 | 3 | 789 | 87.4 | 6.11 | 11.18 |
| P21956 | Mfge8 | 12 | 4 | 4 | 4 | 463 | 51.2 | 6.52 | 11.17 |
| P48962 | Slc25a4 | 21 | 5 | 5 | 2 | 298 | 32.9 | 9.72 | 11.13 |
| Q60930 | Vdac2 | 19 | 4 | 4 | 4 | 295 | 31.7 | 7.49 | 10.88 |
| P51150 | Rab7a | 25 | 4 | 4 | 4 | 207 | 23.5 | 6.7 | 10.84 |
| Q61792 | Lasp1 | 15 | 4 | 4 | 4 | 263 | 30 | 7.05 | 10.83 |
| P51881 | Slc25a5 | 19 | 5 | 5 | 2 | 298 | 32.9 | 9.73 | 10.82 |
| Q9R0E1 | Plod3 | 7 | 4 | 4 | 4 | 741 | 84.9 | 6.23 | 10.69 |
| Q8BH04 | Pck2 | 8 | 4 | 4 | 4 | 640 | 70.5 | 7.28 | 10.18 |
| P00405 | Mtco2 | 16 | 3 | 4 | 3 | 227 | 26 | 4.73 | 9.67 |
| P61027 | Rab10 | 17 | 3 | 4 | 1 | 200 | 22.5 | 8.38 | 9.5 |
| P61620 | Sec61a1 | 9 | 3 | 4 | 3 | 476 | 52.2 | 8.06 | 9.39 |
| Q9DB20 | Atp5po | 17 | 3 | 3 | 3 | 213 | 23.3 | 9.99 | 9.24 |
| Q8VEM8 | Slc25a3 | 11 | 3 | 3 | 3 | 357 | 39.6 | 9.26 | 9.12 |
| Q64523 | H2ac20 | 20 | 2 | 3 | 2 | 129 | 14 | 10.9 | 9.08 |
| Q3U9G9 | Lbr | 7 | 3 | 3 | 3 | 626 | 71.4 | 9.36 | 8.97 |
| P62984 | Uba52 | 20 | 2 | 3 | 2 | 128 | 14.7 | 9.83 | 8.94 |
| Q9CZU6 | Cs | 8 | 3 | 3 | 3 | 464 | 51.7 | 8.57 | 8.79 |
| Q8VDP6 | Cdipt | 22 | 4 | 4 | 4 | 213 | 23.6 | 8.27 | 8.67 |
| P55096 | Abcd3 | 7 | 3 | 3 | 3 | 659 | 75.4 | 9.26 | 8.52 |
| Q9D3H2 | Obp1a | 23 | 3 | 3 | 3 | 163 | 18.5 | 5.67 | 8.05 |
| P51660 | Hsd17b4 | 5 | 3 | 3 | 3 | 735 | 79.4 | 8.57 | 7.95 |
| P15948 | Klk1b22 | 8 | 1 | 2 | 1 | 259 | 28.4 | 6.65 | 7.74 |
| O08573 | Lgals9 | 10 | 3 | 3 | 3 | 353 | 40 | 9.31 | 7.71 |
| Q9JM71 | Klk1b27 | 8 | 1 | 2 | 1 | 263 | 28.7 | 8.56 | 7.4 |
| Q6PHZ2 | Camk2d | 8 | 3 | 3 | 2 | 499 | 56.3 | 7.25 | 7.3 |
| Q61235 | Sntb2 | 7 | 3 | 3 | 3 | 520 | 56.3 | 8.69 | 7.03 |
| P49817 | Cav1 | 18 | 3 | 3 | 3 | 178 | 20.5 | 6.02 | 6.98 |
| P67778 | Phb | 12 | 3 | 3 | 3 | 272 | 29.8 | 5.76 | 6.94 |
| O35474 | Edil3 | 9 | 3 | 3 | 3 | 480 | 53.7 | 7.58 | 6.94 |
| Q91V61 | Sfxn3 | 13 | 2 | 2 | 2 | 321 | 35.4 | 9.51 | 6.89 |
| P35486 | Pdha1 | 8 | 3 | 3 | 3 | 390 | 43.2 | 8.19 | 6.79 |
| O08547 | Sec22b | 14 | 3 | 3 | 3 | 215 | 24.7 | 8.51 | 6.68 |
| Q01149 | Col1a2 | 3 | 3 | 3 | 3 | 1372 | 129.5 | 9.19 | 6.67 |
| P00757 | Klk1b4 | 9 | 1 | 2 | 1 | 256 | 28.5 | 4.86 | 6.24 |
| P01872 | Ighm | 6 | 2 | 2 | 2 | 454 | 49.9 | 7.01 | 6.08 |
| Q9EQQ2 | Yipf5 | 8 | 2 | 2 | 2 | 257 | 27.9 | 4.36 | 5.76 |
| P08228 | Sod1 | 15 | 2 | 2 | 2 | 154 | 15.9 | 6.51 | 5.62 |
| G3X9C2 | Nccrp1 | 9 | 2 | 2 | 2 | 266 | 30.4 | 7.12 | 5.59 |
| Q99LC3 | Ndufa10 | 9 | 2 | 2 | 2 | 355 | 40.6 | 7.78 | 5.56 |
| Q9CY27 | Tecr | 7 | 2 | 2 | 2 | 308 | 36.1 | 9.55 | 5.53 |
| P01027 | C3 | 2 | 2 | 2 | 2 | 1663 | 186.4 | 6.73 | 5.47 |
| Q99MR8 | Mccc1 | 4 | 2 | 2 | 2 | 717 | 79.3 | 7.83 | 5.41 |
| P29788 | Vtn | 6 | 2 | 2 | 2 | 478 | 54.8 | 5.88 | 5.29 |
| Q8C145 | Slc39a6 | 5 | 2 | 2 | 2 | 765 | 86.3 | 6.84 | 5.23 |
| Q9CR67 | Tmem33 | 9 | 2 | 2 | 2 | 247 | 28 | 9.66 | 5.15 |
| Q9Z210 | Pex11b | 5 | 2 | 2 | 2 | 259 | 28.7 | 9.95 | 5.15 |
| Q8R0S2 | Iqsec1 | 2 | 2 | 2 | 2 | 961 | 107.9 | 7.14 | 5.07 |
| Q08189 | Tgm3 | 2 | 1 | 2 | 1 | 693 | 77.3 | 6.81 | 5.05 |
| Q922Q8 | Lrrc59 | 8 | 2 | 2 | 2 | 307 | 34.9 | 9.52 | 4.98 |
| P01831 | Thy1 | 15 | 2 | 2 | 2 | 162 | 18.1 | 8.97 | 4.95 |
| P62071 | Rras2 | 11 | 2 | 2 | 2 | 204 | 23.4 | 6.01 | 4.85 |
| Q99JY0 | Hadhb | 4 | 2 | 2 | 2 | 475 | 51.4 | 9.38 | 4.8 |
| P70227 | Itpr3 | 1 | 2 | 2 | 2 | 2670 | 304.1 | 6.54 | 4.71 |
| Q9JIS8 | Slc12a4 | 2 | 2 | 2 | 2 | 1085 | 120.5 | 6.67 | 4.45 |
| P11088 | Flg | 7 | 1 | 1 | 1 | 336 | 35.7 | 10.6 | 4.41 |
| P24369 | Ppib | 10 | 2 | 2 | 2 | 216 | 23.7 | 9.55 | 4.35 |
| Q9DBS1 | Tmem43 | 5 | 2 | 2 | 2 | 400 | 44.8 | 7.36 | 4.1 |
| P26041 | Msn | 3 | 2 | 2 | 2 | 577 | 67.7 | 6.6 | 4.1 |
| Q9DCF9 | Ssr3 | 8 | 1 | 1 | 1 | 185 | 21.1 | 9.61 | 3.9 |
| Q99K23 | Ufsp2 | 4 | 1 | 1 | 1 | 461 | 52.5 | 6.76 | 3.84 |
| P20801 | Tnnc2 | 10 | 1 | 1 | 1 | 160 | 18.1 | 4.2 | 3.48 |
| P17897 | Lyz1 | 8 | 1 | 1 | 1 | 148 | 16.8 | 9.41 | 3.44 |
| Q9JKN1 | Slc30a7 | 4 | 1 | 1 | 1 | 378 | 41.8 | 6.84 | 3.37 |
| P15947 | Klk1 | 8 | 1 | 1 | 1 | 261 | 28.8 | 5.12 | 3.25 |
| P04071 | Klk1b16 | 5 | 1 | 1 | 1 | 261 | 28.7 | 5.64 | 3.18 |
| O08734 | Bak1 | 5 | 1 | 1 | 1 | 209 | 23.3 | 6.18 | 3.15 |
| P57787 | Slc16a3 | 3 | 1 | 1 | 1 | 470 | 50.3 | 7.96 | 3.09 |
| Q99L04 | Dhrs1 | 4 | 1 | 1 | 1 | 313 | 34 | 8.35 | 3.08 |
| P16110 | Lgals3 | 4 | 1 | 1 | 1 | 264 | 27.5 | 8.38 | 2.97 |
| Q64310 | Surf4 | 6 | 1 | 1 | 1 | 269 | 30.4 | 7.78 | 2.96 |
| P15946 | Klk1b11 | 8 | 1 | 1 | 1 | 261 | 28.7 | 7.14 | 2.87 |
| D3Z7P3 | Gls | 2 | 1 | 1 | 1 | 674 | 73.9 | 7.99 | 2.87 |
| O08917 | Flot1 | 3 | 1 | 1 | 1 | 428 | 47.5 | 7.15 | 2.84 |
| Q8BFR5 | Tufm | 3 | 1 | 1 | 1 | 452 | 49.5 | 7.56 | 2.82 |
| P01642 | Gm10881 | 10 | 1 | 1 | 1 | 115 | 12.6 | 5.94 | 2.79 |
| P18572 | Bsg | 3 | 1 | 1 | 1 | 389 | 42.4 | 5.85 | 2.79 |
| Q9Z0R9 | Fads2 | 4 | 1 | 1 | 1 | 444 | 52.4 | 8.82 | 2.73 |
| P84096 | Rhog | 10 | 1 | 1 | 1 | 191 | 21.3 | 8.12 | 2.68 |
| P00756 | Klk1b3 | 8 | 1 | 1 | 1 | 261 | 29 | 6.84 | 2.68 |
| P53986 | Slc16a1 | 3 | 1 | 1 | 1 | 493 | 53.2 | 7.47 | 2.66 |
| P86048 | Rpl10l | 6 | 1 | 1 | 1 | 214 | 24.5 | 10.1 | 2.64 |
| Q6ZQI3 | Mlec | 4 | 1 | 1 | 1 | 291 | 32.3 | 6.05 | 2.63 |
| Q5FW60 | Mup20 | 6 | 1 | 1 | 1 | 181 | 20.9 | 4.89 | 2.61 |
| Q91VR2 | Atp5f1c | 3 | 1 | 1 | 1 | 298 | 32.9 | 9.01 | 2.57 |
| P08071 | Ltf | 1 | 1 | 1 | 1 | 707 | 77.8 | 8.53 | 2.55 |
| Q99JR1 | Sfxn1 | 4 | 1 | 1 | 1 | 322 | 35.6 | 9.23 | 2.54 |
| Q91Z96 | Bmp2k | 1 | 1 | 1 | 1 | 1138 | 126.1 | 6.8 | 2.54 |
| Q6NWW9 | Fndc3b | 2 | 1 | 1 | 1 | 1207 | 132.7 | 6.29 | 2.53 |
| Q8BK08 | Tmem11 | 8 | 1 | 1 | 1 | 190 | 21.3 | 7.36 | 2.51 |
| O55022 | Pgrmc1 | 7 | 1 | 1 | 1 | 195 | 21.7 | 4.7 | 2.51 |
| Q8JZU2 | Slc25a1 | 4 | 1 | 1 | 1 | 311 | 33.9 | 9.89 | 2.49 |
| O70378 | Emc8 | 4 | 1 | 1 | 1 | 207 | 23.3 | 6.15 | 2.49 |
| Q99JH8 | Kdelr1 | 9 | 1 | 1 | 1 | 212 | 24.5 | 8.62 | 2.48 |
| Q920L1 | Fads1 | 2 | 1 | 1 | 1 | 447 | 52.3 | 9.29 | 2.46 |
| Q8C650 | Septin10 | 2 | 1 | 1 | 1 | 452 | 52.4 | 6.6 | 2.45 |
| Q8BHC4 | Dcakd | 5 | 1 | 1 | 1 | 231 | 26.5 | 9.58 | 2.44 |
| Q91ZN5 | Slc35b2 | 2 | 1 | 1 | 1 | 431 | 47.3 | 9.29 | 2.43 |
| Q91XB7 | Yif1a | 5 | 1 | 1 | 1 | 293 | 32.1 | 9.09 | 2.42 |
| Q9JJE7 | Fads3 | 3 | 1 | 1 | 1 | 449 | 51.4 | 7.49 | 2.42 |
| P04939 | Mup3 | 6 | 1 | 1 | 1 | 184 | 21.5 | 4.81 | 2.41 |
| Q9QYF1 | Rdh11 | 4 | 1 | 1 | 1 | 316 | 35.1 | 8.91 | 2.39 |
| Q9QYA2 | Tomm40 | 3 | 1 | 1 | 1 | 361 | 37.9 | 7.74 | 2.38 |
| Q9JLF6 | Tgm1 | 1 | 1 | 1 | 1 | 815 | 89.8 | 6.51 | 2.35 |
| Q61495 | Dsg1a | 1 | 1 | 1 | 1 | 1057 | 114.5 | 4.89 | 2.34 |
| Q05793 | Hspg2 | 0 | 1 | 1 | 1 | 3707 | 398 | 6.32 | 2.33 |
| O35682 | Myadm | 3 | 1 | 1 | 1 | 320 | 35.3 | 8.31 | 2.32 |
| Q9CRD2 | Emc2 | 3 | 1 | 1 | 1 | 297 | 34.9 | 6.81 | 2.32 |
| Q3V3R1 | Mthfd1l | 1 | 1 | 1 | 1 | 977 | 105.7 | 7.02 | 2.31 |
| P01633 | Igk-V19-17 | 6 | 1 | 1 | 1 | 149 | 16.4 | 6.92 | 2.31 |
| P84091 | Ap2m1 | 2 | 1 | 1 | 1 | 435 | 49.6 | 9.54 | 2.29 |
| Q8BMD8 | Slc25a24 | 3 | 1 | 1 | 1 | 475 | 52.9 | 7.43 | 2.26 |
| Q5PR73 | Diras2 | 6 | 1 | 1 | 1 | 199 | 22.5 | 8.76 | 2.25 |
| Q8BU31 | Rap2c | 6 | 1 | 1 | 1 | 183 | 20.7 | 4.94 | 2.23 |
| Q9EST1 | Gsdma | 2 | 1 | 1 | 1 | 446 | 49.6 | 5.57 | 2.23 |
| P97300 | Nptn | 3 | 1 | 1 | 1 | 397 | 44.3 | 7.74 | 2.16 |
| Q61176 | Arg1 | 3 | 1 | 1 | 1 | 323 | 34.8 | 7.01 | 2.13 |
| Q8QZT1 | Acat1 | 3 | 1 | 1 | 1 | 424 | 44.8 | 8.51 | 2.13 |
| Q9D051 | Pdhb | 3 | 1 | 1 | 1 | 359 | 38.9 | 6.87 | 2.09 |
| Q9JLJ5 | Elovl1 | 5 | 1 | 1 | 1 | 279 | 32.7 | 9.63 | 2.09 |
| Q9CQ62 | Decr1 | 4 | 1 | 1 | 1 | 335 | 36.2 | 8.95 | 2.06 |
| Q9DC23 | Dnajc10 | 1 | 1 | 1 | 1 | 793 | 90.5 | 6.96 | 2.05 |
| Q99KI0 | Aco2 | 1 | 1 | 1 | 1 | 780 | 85.4 | 7.93 | 2.04 |
| O70579 | Slc25a17 | 3 | 1 | 1 | 1 | 307 | 34.4 | 10.1 | 2.03 |
| Q7SIG6 | Asap2 | 1 | 1 | 1 | 1 | 958 | 106.7 | 6.65 | 2.01 |
| Q8R1L4 | Kdelr3 | 4 | 1 | 1 | 1 | 214 | 25.1 | 9.06 | 2 |
| P84244 | H3-3a | 8 | 1 | 1 | 1 | 136 | 15.3 | 11.3 | 2 |
| P61804 | Dad1 | 11 | 1 | 1 | 1 | 113 | 12.5 | 7.08 | 1.98 |
| Q8K009 | Aldh1l2 | 2 | 1 | 1 | 1 | 923 | 101.5 | 6.29 | 1.98 |
| Q9CQQ7 | Atp5pb | 3 | 1 | 1 | 1 | 256 | 28.9 | 9.06 | 1.97 |
| Q3TXX3 | Zfyve27 | 1 | 1 | 1 | 1 | 415 | 46.2 | 5.35 | 1.96 |
| P45591 | Cfl2 | 7 | 1 | 1 | 1 | 166 | 18.7 | 7.88 | 1.94 |
| O09172 | Gclm | 3 | 1 | 1 | 1 | 274 | 30.5 | 5.52 | 1.92 |
| Q9R0M6 | Rab9a | 5 | 1 | 1 | 1 | 201 | 22.9 | 5.66 | 1.91 |
| P11438 | Lamp1 | 3 | 1 | 1 | 1 | 406 | 43.8 | 8.4 | 1.91 |
| Q99JB2 | Stoml2 | 4 | 1 | 1 | 1 | 353 | 38.4 | 8.87 | 1.9 |

Table S3 Primers for genotyping and knockout efficiency.

| Gene | Application | Primer sequence（5'→ 3'） |
| --- | --- | --- |
| NELL2 forward | genotyping | GGCAGATGGGTGATTTGGG |
| NELL2 reverse | genotyping | GGAGCGCTCCATGGTAACC |
| Col1-cre forward | genotyping | CCCAGCTCTCCATCAAGATG |
| Col1-cre reverse | genotyping | GTGAAACAGCATTGCTGTCACTT |
| NELL2 forward | knockout efficiency | CTCCCGAATTTAAGTGGATCTG |
| NELL2 reverse | knockout efficiency | GGACTGCATAATGGGACGAA |

Table S4 Primers for cloning.

| Cloning primers | Primer sequence（5'→ 3'） |
| --- | --- |
| Nell2 d32-217F | TAGGGAGGGGTCCACACCAA |
| Nell2 d32-217R | GTGGACCCCTCCCTACAGGGCTTCATTGCTCAGTG |
| Nell2 d242-271F | GCTCACAGTACACAAGCCCATGGAAGTCGTTG |
| Nell2 d242-271R | TTCCATGGGCTTGTGTACTGTGAGCGGACATGCAC |
| Nell2 d277-398F | TGTCCGCTCACAGTAGCACT |
| Nell2 d277-398R | TACTGTGAGCGGACAAAAGGTTATGACTTCTGTTC |
| Nell2 d406-644F | CCTCGTGAGAACAGAAGTCATAACCTTTAC |
| Nell2 d406-644R | TATGACTTCTGTTCTCACGAGGGGAAAGTGAAGCA |
| Nell2 d643-758F | GTCCCCAGTGCAGTTCTTCC |
| Nell2 d643-758R | AACTGCACTGGGGACGTCACCGATCCTTGTCAGGC |
| Fn1-NheI-F1 | AGACCCAAGCTGGCTAGGCCACCATGCTCAGGGGTCCGGGACCC |
| Fn1-R1 | GATAGTGATGTTGTACTGAACAC |
| Fn1-F2 | CAGTACAACATCACTATCTATGCTGTGGAGGAGAACCAGG |
| Fn1-R2 | GTTGGTCACTGCAGTTTGAAC |
| Fn1-F3 | CAAACTGCAGTGACCAACATTGATCGCCCTAAAGGACTGG |
| Fn1-XbaI-R3 | TTTAAACGGGCCCTCTAGTTAAGCGTAATCTGGAACATCGTATGGGTACTCTCGAGAATCGTCTCTGT |
| Fn1 d53-347F | GCCAGGCTTGCTCTGACTGA |
| Fn1 d53-347R | CAGAGCAAGCCTGGCACCCAGACTTATGGTGGCAA |
| Fn1 d353-461F | ACCATAAGTCTGGGTCACGG |
| Fn1 d353-461R | ACCCAGACTTATGGTTGCCCAATGGCTGCCCACGA |
| Fn1 d470-604F | GATCTCCTCGTGGGCAGCCA |
| Fn1 d470-604R | GCCCACGAGGAGATCTACCCAGGCACAACTGGACC |
| Fn1 d609-1160F | CATCTCTTGTGCCTGGGTAGGTCTGCA |
| Fn1 d609-1160R | ACCTACCCAGGCACAAGAGATGCACCGATTGTCAA |
| Fn1 d1173-1711F | CGGTGTCACTACTCTGTTGA |
| Fn1 d1173-1711R | AGAGTAGTGACACCGCAGCCCCTGGTTCAAACTGC |
| Fn1 d1722-2272F | GTTGGTCACTGCAGTTTGAA |
| Fn1 d1722-2272R | ACTGCAGTGACCAACGTTCGGGAAGAGGTTGTGAC |
| Fn1 d2296-2425F | TGAGTCATCTGTAGGCTGGT |
| Fn1 d2296-2425R | CCTACAGATGACTCACCTGGGGCTGCTGAACCCAG |
| Fn1 C-terminal-Frag1R | CTGACAGCCACCGGGGATTGAGGCTGCA |
| Fn1 C-terminal-Frag2F | CCCCGGTGGCTGTCAGCGAAGGCCTGAA |

Table S5 Primers for qRT-PCR.

| Gene | Primer sequence（5'→ 3'） |
| --- | --- |
| Fn1 forward | CCCTATCTCTGATACCGTTGTCC |
| Fn1 reverse | TGCCGCAACTACTGTGATTCGG |
| NELL2 forward | GAACCACCTACCGAGAGTCTGA |
| NELL2 reverse | CTCCTTACAGCACTTGCCATCC |
| NELL1 forward | TGCCCTCCTTTGAACTGCTCAG |
| NELL1 reverse | CCACACTTAGGTGCTTCTGCAC |
| Actb forward | CATTGCTGACAGGATGCAGAAGG |
| Actb qRTR | TGCTGGAAGGTGGACAGTGAGG |
| PPARγ forward | CTTGACAGGAAAGACAACGG |
| PPARγ reverse | GCTTCTACGGATCGAAACTG |
| C/EBPα forward | CTGATTCTTGCCAAACTGAG |
| C/EBPα reverse | GAGGAAGCTAAGACCCACTAC |
| aP2 forward | AAATCACCGCAGACGACAGG |
| aP2 reverse | GGCTCATGCCCTTTCATAAAC |
| Sp7 forward | GGCTTTTCTGCGGCAAGAGGTT |
| Sp7 reverse | CGCTGATGTTTGCTCAAGTGGTC |
| Alp forward | CCAGAAAGACACCTTGACTGTGG |
| Alp reverse | TCTTGTCCGTGTCGCTCACCAT |
| Opn forward | GCTTGGCTTATGGACTGAGGTC |
| Opn reverse | CCTTAGACTCACCGCTCTTCATG |
| Bsp forward | GAGCCAGGACTGCCGAAAGGA |
| Bsp reverse | CCGTTGTCTCCTCCGCTGCTGC |
| Runx2 forward | CCTGAACTCTGCACCAAGTCCT |
| Runx2 reverse | TCATCTGGCTCAGATAGGAGGG |
| Opg forward | CGGAAACAGAGAAGCCACGCAA |
| Opg reverse | CTGTCCACCAAAACACTCAGCC |
| RANKL forward | GTGAAGACACACTACCTGACTCC |
| RANKLreverse | GCCACATCCAACCATGAGCCTT |
| Acp5 forward | GCGACCATTGTTAGCCACATACG |
| Acp5 reverse | CGTTGATGTCGCACAGAGGGAT |
| Ctsk forward | AGCAGAACGGAGGCATTGACTC |
| Ctsk reverse | CCCTCTGCATTTAGCTGCCTTTG |
| Nfatc1 forward | GGTGCCTTTTGCGAGCAGTATC |
| Nfatc1 reverse | CGTATGGACCAGAATGTGACGG |
| Mmp9 forward | GCTGACTACGATAAGGACGGCA |
| Mmp9 reverse | TAGTGGTGCAGGCAGAGTAGGA |
| Fn1 C-terminal forward | CGAAGCCGGGAAGAGCAAGA |
| Fn1 C-terminal reverse | TGGCGTAATGGGAAACCGTGTA |

Table S6 Antibody list.

| Antibodies | Source | Identifier |
| --- | --- | --- |
| Rabbit monoclonal anti-PPARγ | Cell Signaling Technology, MA, USA | Cat#2435 |
| Rabbit polyclonal anti-C/EBPa | Cell Signaling Technology, MA, USA | Cat#2295 |
| Rabbit monoclonal anti-Runx2 | Cell Signaling Technology, MA, USA | Cat#12556 |
| Rabbit monoclonal anti-Phospho-Akt (Ser473) | Cell Signaling Technology, MA, USA | Cat#4060 |
| Rabbit polyclonal anti-FAK | Cell Signaling Technology, MA, USA | Cat#3285 |
| Rabbit monoclonal anti-ALP | Huabio, Hangzhou, China | Cat#ET1601-21 |
| Rabbit polyclonal anti-osterix | Affinity Biosciences, OH.USA | Cat#DF7731 |
| Rabbit polyclonal anti-Phospho-FAK (Tyr397) | Affinity Biosciences, OH.USA | Cat#AF3398 |
| Rabbit polyclonal anti-AKT1 | Affinity Biosciences, OH.USA | Cat#AF0836 |
| Rabbit polyclonal anti-NELL2 | Abclonal, Wuhan, China | Cat#A19327 |
| Rabbit polyclonal anti-Fn1 | Proteintech, Wuhan, China | Cat#12613-a-AP |
| Rabbit polyclonal anti-ITGB1 | Proteintech, Wuhan, China | Cat#12594-1-AP |
| Mouse monoclonal anti-β-actin | Proteintech, Wuhan, China | Cat#66009-1-Ig |
| Mouse monoclonal anti-GAPDH | Proteintech, Wuhan, China | Cat#60004-1-Ig |
| Rabbit polyclonal anti-aP2/FABP4 | Proteintech, Wuhan, China | Cat#12802-1-AP |
| Rabbit anti-Osteopontin | Abclonal, Wuhan, China | Cat#A19092 |
| Goat anti-mouse IgG(H+L)-HRP | Simu, Tianjin, China | Cat#S2002 |
| Goat anti-Rabbit IgG(H+L)-HRP | Sungene, Tianjin, China | Cat#LK2001 |
